# Preparation of a First ^18^F-Labeled Agonist for M_1_ Muscarinic Acetylcholine Receptors

**DOI:** 10.3390/molecules25122880

**Published:** 2020-06-23

**Authors:** Boris D. Zlatopolskiy, Felix Neumaier, Till Rüngeler, Birte Drewes, Niklas Kolks, Bernd Neumaier

**Affiliations:** 1 Institute of Neuroscience and Medicine, Nuclear Chemistry (INM-5), Forschungszentrum Jülich GmbH, 52428 Jülich, Germany; boris.zlatopolskiy@uk-koeln.de (B.D.Z.); b.drewes@fz-juelich.de (B.D.); niklas.kolks@uk-koeln.de (N.K.); 2 Institute of Radiochemistry and Experimental Molecular Imaging, University Clinic Cologne, 50931 Cologne, Germany; till@ruengeler.eu; 3 Max Planck Institute for Metabolism Research, 50931 Cologne, Germany; 4 Institute of Neurophysiology, University Hospital Cologne, Robert-Koch Str. 39, 50931 Cologne, Germany; felix@neumaier-net.de

**Keywords:** radiolabeling, fluorine-18, positron emission tomography, acetylcholine receptor agonist, Cu-mediated radiofluorination

## Abstract

M_1_ muscarinic acetylcholine receptors (mAChRs) are abundant in postsynaptic nerve terminals of all forebrain regions and have been implicated in the cognitive decline associated with Alzheimer’s disease and other CNS pathologies. Consequently, major efforts have been spent in the development of subtype-selective positron emission tomography (PET) tracers for mAChRs resulting in the development of several ^11^C-labeled probes. However, protocols for the preparation of ^18^F-labeled mAChR-ligands have not been published so far. Here, we describe a straightforward procedure for the preparation of an ^18^F-labeled M_1_ mAChR agonist and its corresponding pinacol boronate radiolabeling precursor and the non-radioactive reference compound. The target compounds were prepared from commercially available aryl fluorides and Boc protected 4-aminopiperidine using a convergent reaction protocol. The radiolabeling precursor was prepared by a modification of the Miyaura reaction and labeled via the alcohol-enhanced Cu-mediated radiofluorination. The developed procedure afforded the radiotracer in a non-decay-corrected radiochemical yield of 17 ± 3% (*n* = 3) and in excellent radiochemical purity (>99%) on a preparative scale. Taken together, we developed a straightforward protocol for the preparation of an ^18^F-labeled M_1_ mAChR agonist that is amenable for automation and thus provides an important step towards the routine production of a ^18^F-labeled M_1_ selective PET tracer for experimental and diagnostic applications.

## 1. Introduction

**Scheme 1:**
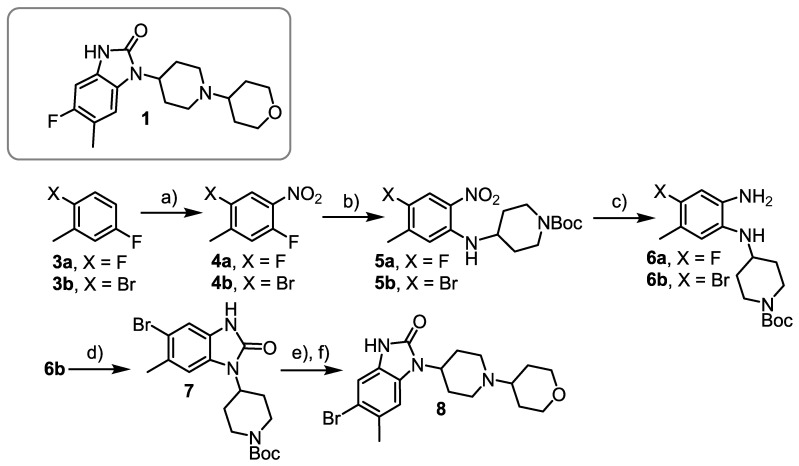
Preparation of intermediates **6a** and **8** according to the modified protocol of Budzik et al. [9]. Conditions: (**a**) KNO_3_, H_2_SO_4_, 16 h, r.t., 64% and 72% for **4a** and **4b**, respectively; (**b**) 4-amino-*N*-Boc-piperidine, DIEA, DMF, 70–80 °C, 38 h, 63% and 72% for **5a** and **5b**, respectively; (**c**) Raney nickel, N_2_H_4_·H_2_O, EtOH, 45 °C, 2 h, 70% and 29% for **6a** and **6b**, respectively; (**d**) triphosgene, Et_3_N, THF, 45 °C, 2 h, 94%; (**e**) TFA/CH_2_Cl_2_, 1 h or HCl/EtOAc, r.t., 1 h, then (**f**) NaBH_3_CN, Bu_4_NBr, Et_3_N, DMF, r.t., 48 h, 6%. r.t.—room temperature.

Muscarinic acetylcholine receptors (mAChRs) in the central and peripheral nervous system participate in autonomic, cognitive, and motor function. To date, five subtypes (M_1_–M_5_) have been identified, all of which share a highly conserved ligand-binding site for acetylcholine. M_1_ mAChRs are the most prevalent subtype in the CNS and abundant in postsynaptic nerve terminals of all forebrain regions [1,2]. Substantial evidence suggests that they are critically involved in cognition and that loss of cholinergic function contributes to the cognitive decline associated with Alzheimer’s disease, schizophrenia, and other neurological and psychiatric disorders [3,4,5]. These findings have spurred interest into M_1_ receptors as a target for experimental or clinical applications. In this context, functional imaging techniques and especially positron emission tomography (PET) have an enormous potential for in vivo assessment of target engagement [6]. A lot of effort spent in the development of PET tracers for mAChRs led to the discovery of several subtype selective ^11^C-labeled radioligands [7,8]. However, to the best of our knowledge M_1_ receptor subtype selective ^18^F-labeled mAChR ligands have not been reported so far. Herewith we disclose the preparation of the radiofluorinated allosteric M_1_-specific mAChR agonist, 5-[^18^F]fluoro-6-methyl-1-(1-(tetrahydro-2*H*-pyran-4-yl)piperidin-4-yl)-1,3-dihydro-2*H*-benz[d]imida-zole-2-one ([^18^F]**1**), discovered by Budzik et al. [9] (Scheme 1). Ligand **1** has been shown to possess excellent pharmacokinetic properties and robust pro-cognitive activity in animal models [9], making [^18^F]**1** a potentially useful probe for in vivo tracer studies.

## 2. Results and Discussion

### 2.1. Preparation of the Reference Compound **1** and Pinacol Boronate Precursor **8** for Radiolabeling

Initially the modified procedure of Budzik et al. [9] was applied in order to prepare the non-radioactive ligand **1** and the intermediate **8** (Scheme 1). This synthetic route consists of nitration of the respective 2-fluoro-5-halotoluene followed by the selective nucleophilic substitution of the fluorine substituent in ortho-position to the nitro group with 4-*N*-Boc-aminopiperidine, reduction of the nitro group, cyclization to the benzimidazolone, *N*-Boc deprotection and, finally, reductive alkylation of the resulting intermediate with 4-tetrahydropyranone. The nitration step was performed as described by Kher et al. [10], except that the reaction time was increased from 30 min to 16 h, affording known **4a** and **4b** in yields of 64% and 72%, respectively. The subsequent steps furnished the corresponding *o*-phenylendiamines, **6a** and **6b**, in 70% and 29% yield, respectively. Cyclization of **6b** using triphosgene instead of the originally proposed 1,1′-carbonyldiimidazole, afforded benzimidazolone **7** in almost quantitative yield. Unexpectedly, deprotection of this intermediate by HCl in EtOAc or trifluoroacetic acid (neat or in CH_2_Cl_2_) gave rise to a number of by-products. Reductive alkylation of the respective crude amine afforded **8** in only 6% yield.

**Scheme 2:**
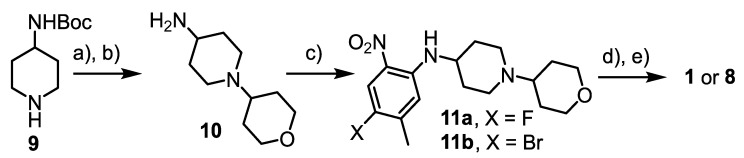
Convergent synthesis of ligand **1** and intermediate **8**. Conditions: (**a**) tetrahydro-4*H*-pyran-4-one, NaBH_3_CN, Bu_4_NBr, Et_3_N, DMF, 36 h, 57%; (**b**) HCl/EtOAc, 1 h, then NaOH, 93%; (**c**) **4a** or **4b**, DIEA, DMF, 70 °C, 16 h, 58% and 99% (crude) for **11a** and **11b**, respectively; (**d**) **11a**, Raney nickel, N_2_H_4_·H_2_O, EtOH, 45 °C, 2 h, 63% or **11b**, NH_4_Cl, Zn, EtOH/EtOAc, 16 h, 90%; (**e**) triphosgene, Et_3_N, THF, 45 °C, 2 h, 60% and 52% for **1** and **8**, respectively.

In order to circumvent the problems mentioned above, 1-(tetrahydro-2*H*-pyran-4-yl)piperidine-4-amine (**10**) was prepared by reductive alkylation of 4-*N*-Boc-aminopiperidine (**9**) with tetrahydro-4*H*-pyran-4-one using NaBH_3_CN as reductant and Bu_4_NBr as phase transfer catalyst followed by *N*-Boc deprotection and was allowed to react with **4a** or **4b** (Scheme 2) affording the corresponding *o*-nitroanilines **11a** and **11b** in good yields. Reduction of the nitro group in **11a** using Raney nickel and hydrazine hydrate proceeded smoothly, furnishing the corresponding *o*-phenylenediamine intermediate in 63% yield. In contrast, application of the same procedure to the bromo-substituted nitroaniline **11b** afforded only traces of the product. Among several reductants tested, powdered Zn/NH_4_Cl in a mixture of EtOH and EtOAc according to the modified procedure of Tsukinoki and Tsuzuki [11] enabled the preparation of the desired intermediate in an excellent (>90%) yield. Subsequent cyclization of the prepared *o*-phenylenediamines with triphosgene afforded ligand **1** and substituted imidazolone **8** in 14% and 33% yield over four steps, respectively.

**Scheme 3:**
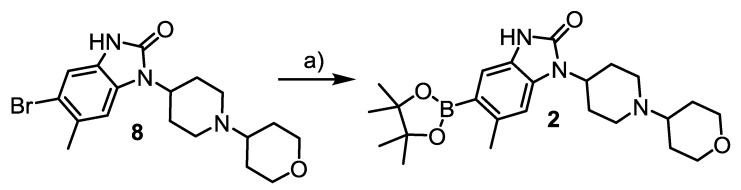
Preparation of the precursor for radiolabeling **2** by Miyaura cross-coupling reaction. Conditions: B_2_Pin_2_, Pd(dppf)Cl_2_, KOAc, 1,4-dioxane, 110 °C, 3 h, 48%.

The boronic acid pinacol ester **2** was prepared in 48% yield by the Miyaura reaction, using a modification of the protocol described by Ishiyama et al. [12] (Scheme 3). The Pd content determined by ICP/MS amounted to 1.2 ± 0.1 ppm.

### 2.2. Preparation of [^18^F]**1**

**Scheme 4:**
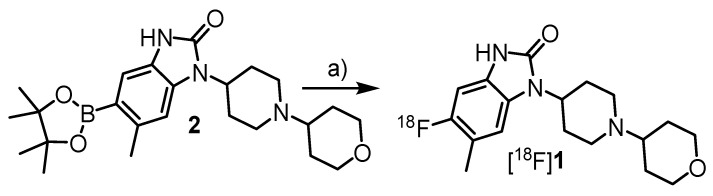
Preparation of [^18^F]**1** via alcohol-enhanced Cu-mediated radiofluorination. Conditions: (**a**) elution of ^18^F^–^ in the form of [^18^F]Et_4_NF with Et_4_NHCO_3_ in MeOH; removal of MeOH; **9**, Cu(py)_4_(OTf)_2_, 2:1 DMA/*n*BuOH, 110 °C, 10 min, air or argon [RCC = 96 ± 3% (*n* = 3)]; HPLC purification; formulation (n.d.c RCY = 13–19%).

**Figure 1:**
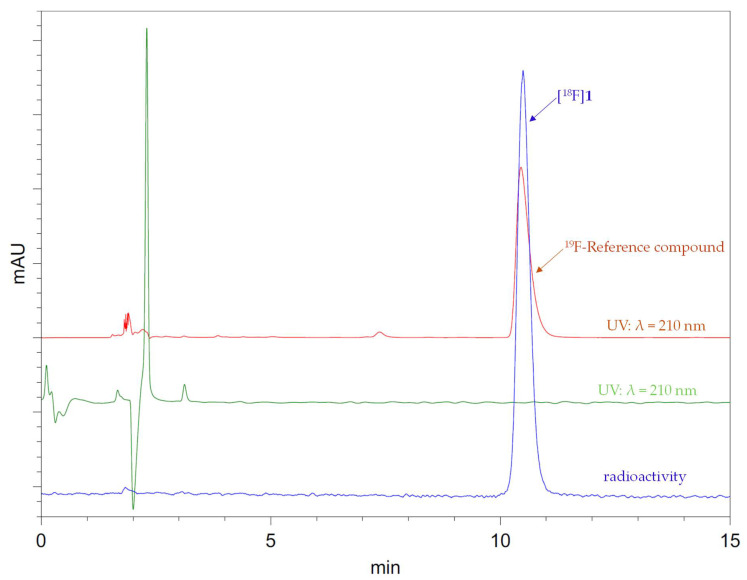
HPLC traces of the purified and formulated radiotracer [^18^F]**1**, and the ^19^F-reference compound **1**. Blue trace: [^18^F]**1** (radioactivity channel); green trace: [^18^F]**1** (UV channel, λ = 210 nm); red trace: ^19^F-reference compound (UV channel, λ = 210 nm).

Radiolabeling of the Bpin ester **2** was performed according to the modified protocol for Cu-mediated alcohol-enhanced radiofluorination [13,14,15,16,17] (Scheme 4). [^18^F]Fluoride was loaded onto an anion exchange resin and eluted with a solution of Et_4_NHCO_3_ in MeOH. After evaporation of MeOH, [^18^F]Et_4_NF/Et_4_NHCO_3_ was taken up in a solution of **2** and Cu(py)_4_(OTf)_2_ in 2:1 DMA/*n*BuOH and the reaction mixture was heated at 110 °C for 10 min under Ar or air to afford the ^18^F-labeled ligand [^18^F]**1** in excellent (>90%) radiochemical conversions (RCCs). The crude tracer obtained after concentration of the reaction mixture under reduced pressure was purified by HPLC and formulated as a ready-to-use solution. On a preparative scale [^18^F]**1** was produced in a non-decay corrected radiochemical yield (n.d.c RCY) of 17 ± 3% (*n* = 3) and in excellent radiochemical purity (>99%) within 90–100 min (Figure 1). Besides the molar activity, which is dependent on the activity amount, the carrier amount per batch was measured (please refer to [18] for further discussion). The latter amounted to 25.2 nmol/batch and the molar activity to 30.8 GBq/µmol (measured for 770 MBq [^18^F]**1**; refer to the Supplementary Materials for more details). The Cu content, measured by ICP/MS, amounted to 3.4 ± 0.1 µg/batch and was below any level of concern according to the ICH Guideline of Elemental Impurities (Q3D) [19].

## 3. Materials and Methods

### 3.1. General

Chemicals and solvents were purchased from Sigma-Aldrich (Steinheim, Germany), Merck KGaA (Darmstadt, Germany), OxChem (Wood Dale, IL, USA), VWR International (Radnor, PA, USA) and Alfa Aesar (Haverell, MA, USA) and used without further purification.

### 3.2. Nuclear Magnetic Resonance Spectroscopy (NMR)

Unless otherwise stated, all NMR-Spectra were measured in CDCl_3_. ^1^H-NMR spectra were obtained with a Bruker DPX Avance 300 (Bruker, Rheinstetten, Germany). ^1^H chemical shifts are reported in ppm relative to residual peaks of deuterated solvents. The observed signal multiplicities are characterized as follows: s = singlet, d = doublet, t = triplet, m = multiplet and q = quartet. Coupling constants are reported in Hertz (Hz). ^13^C-NMR spectra [additional APT (Attached Proton Test)]: Bruker DPX Avance 300 (75 MHz). ^13^C chemical shifts are reported in ppm relative to residual peaks of deuterated solvents. ^1^H-, ^13^C- and ^19^F-NMR spectra are provided in the Supplementary Materials.

### 3.3. Mass Spectroscopy

Mass spectra (MS) were measured with a LTQ Orbitrap XL (Thermo Fisher Scientific Inc., Bremen, Germany).

### 3.4. Chemistry

All reactions were carried out with magnetic stirring. Air or moisture sensitive reagents were handled under argon using either a glovebox or a Schlenk line. Organic extracts were dried over anhydrous MgSO_4_. Solutions were concentrated under reduced pressure at 40–50 °C using a rotary evaporator (Bruker, Rheinstetten, Germany).

Column chromatography was performed with silica gel (w/Ca, 0.1–0.3%), 60Å, 230–400 mesh particle size from Sigma-Aldrich GmbH (Steinheim, Germany). Solvent proportions are indicated in a volume/volume ratio.

Thin layer chromatography (TLC) was performed using aluminium sheets coated with silica gel 60 F_254_ (Merck KGaA, Darmstadt, Germany). Chromatograms were inspected under UV light (λ = 254 nm) and stained with molybdophosphoric acid (10% in ethanol), ninhydrin (0.2% in ethanol) or Dragendorff reagent.

#### 3.4.1. 2,5-Difluoro-4-nitrotoluene (**4a**)

KNO_3_ (3.2 g, 31.2 mmol) was added to an ice-cold solution of 2,5-difluorotoluene (**3a**) (4 g, 31.2 mmol) in concentrated H_2_SO_4_ (15 mL), the mixture was allowed to warm to ambient temperature and stirred at 28 °C overnight. The reaction mixture was then poured over ice and the resulting suspension was extracted with EtOAc (3 × 50 mL). The combined organic layers were concentrated under reduced pressure and the residue was purified by column chromatography (EtOAc/hexane = 1:15) to afford the title compound **4a** [9]. Yield: 3.45 g, 20 mmol (64%). Appearance: yellow crystalline solid [9,10]. ^1^H-NMR: 7.78 (dd, *J* = 8.4, 6.3 Hz, 1H), 7.16 (dd, *J* = 10.9, 6.1 Hz, 1H), 2.39 (d, *J* = 1.8 Hz, 3H).

#### 3.4.2. *tert*-Butyl 4-[(4-fluoro-5-methyl-2-nitrophenyl)amino] piperidine-1-carboxylate (**5a**)

Diisopropylethylamine (0.87 mL, 0.65g, 5 mmol, 1 eq) was added to a solution of **4a** (0.87 g, 5 mmol, 1 eq) and 4-amino-1-*N*-Boc-piperidine (1 g, 5 mmol, 1 eq) in DMF (8 mL) at 40 °C and the resulting solution was stirred at 80 °C for 38 h. After removal of the solvent under reduced pressure and addition of water, the resulting mixture was extracted with CH_2_Cl_2_ (3 × 40 mL). The combined organic fractions were dried and concentrated under reduced pressure. The residue was purified by column chromatography (EtOAc/hexane = 1:15) to afford the title compound **5a** [9]. Yield: 1.12 g, 3.16 mmol (63%). Appearance: orange-red solid. ^1^H-NMR: 8.02 (br, 1H), 7.84 (d, *J* = 10.0 Hz, 1H), 6.67 (d, *J* = 6.3 Hz, 1H), 4.21–3.85 (m, 2H), 3.79–3.55 (m, 1H), 3.08 (t, *J* = 11.1 Hz, 2H), 2.32 (s, 3H), 2.15–1.97 (m, 2H), 1.63–1.52 (m, 2H), 1.49 (s, 9H). ^13^C-NMR: 169.52, 152.27 (d, *J* = 353.3 Hz), 153.07, 141.35, 115.32 (d, *J* = 3.0 Hz), 111.70 (d, *J* = 27.0 Hz), 79.91, 49.40, 42.09, 36.47, 31.78, 28.41.

#### 3.4.3. *tert*-Butyl 4-([2-amino-4-fluoro-5-methylphenyl]amino)piperidine-1-carboxylate

Hydrazine hydrate (700 µL, 680 mg, 14 mmol, 10 eq) was slowly added to a suspension of Raney nickel (0.7 mL, 50% aq. suspension) in a solution of **5a** (0.5 g, 1.4 mmol, 1 eq) in EtOH (30 mL). The mixture was stirred at 45 °C for 2 h and filtered over Celite. After removal of volatiles under reduced pressure and addition of saturated NaHCO_3_ (30 mL), the resulting emulsion was extracted with CH_2_Cl_2_ (3 × 30 mL). The combined organic layers were dried and concentrated under reduced pressure. The residue was purified by column chromatography (EtOAc/hexane = 1:1) followed by crystallization from hexane to afford the title compound *tert*-butyl-4-([2-amine-4-fluoro-5-methylphenyl]amine)piperidine-1-carboxylate [9]. Yield: 0.32 g, 0.98 mmol (70%). Appearance: brown solid. ^1^H-NMR: 6.47 (d, *J* = 7.5 Hz, 1H), 6.42 (d, *J* = 10.5 Hz, 1H, H-6′), 4.10 (d, *J* = 7.2 Hz, 1H), 4.03 (d, *J* = 11.8 Hz, 2H), 3.45 (t, *J* = 13.4 Hz, 2H), 3.25 (ddd, *J* = 13.8, 9.9, 3.7 Hz, 1H), 2.91 (t, *J* = 11.4 Hz, 2H), 2.15 (s, 3H), 1.97 (dd, *J* = 9.1, 3.7 Hz, 2H), 1.47 (s, 9H), 1.41–1.29 (m, 2H). ^13^C-NMR: 157.29, 154.49 (d, *J* = 48.0 Hz), 135.75 (d, *J* = 9.8 Hz), 130.64, 118.46 (d, *J* = 5.7 Hz), 103.66 (d, *J* = 26.1 Hz), 79.54, 51.24, 32.58, 28.44, 22.65, 14.06.

#### 3.4.4. 2-Bromo-5-fluoro-4-nitrotoluene (**4b**)

KNO_3_ (2 g, 20 mmol, 1 eq) was added to an ice-cold solution of **3b** (3.78 g, 20 mmol, 1 eq) in concentrated H_2_SO_4_ (15 mL). The mixture was allowed to reach ambient temperature and stirred overnight. Afterwards, the reaction mixture was poured over ice and extracted with EtOAc (3 × 50 mL). The combined organic fractions were dried, concentrated under reduced pressure and the remaining brown-red oil purified by column chromatography (CH_2_Cl_2_/MeOH = 9:1) to afford the title compound **4b** [10]. Yield: 4.25 g, 14.4 mmol (72%). Appearance: yellow oil. ^1^H-NMR: 8.14 (d, *J* = 7.1 Hz, 1H), 7.18 (d, *J* = 11.4 Hz, 1H), 2.44 (s, 3H).

#### 3.4.5. *tert*-Butyl 4-[(4-bromo-5-methyl-2-nitrophenyl)amino]piperidine-1-carboxylate (**5b**)

DIEA (0.87 mL, 0.65 g, 5 mmol, 1 eq) was added to a solution of **4b** (1.5 g, 5 mmol, 1 eq) and 4-amino-1-*N*-Boc-piperidine (1 g, 5 mmol, 1 eq) in DMF (8 mL) at 40 °C. The resulting mixture was stirred at 80 °C for 38 h and concentrated under reduced pressure. H_2_O (40 mL) was added to the oily residue and the resulting suspension was extracted with CH_2_Cl_2_ (3 × 30 mL). The combined organic fractions were dried and concentrated under reduced pressure. The residue was purified by column chromatography (EtOAc/hexane = 1:15) to afford the title compound. Yield: 1.6 g, 3.6 mmol (72%). Appearance: orange solid. ^1^H-NMR: (300 MHz, CDCl_3_) δ (ppm) = 8.35 (s, 1H), 8.06 (br, 1H), 6.74 (s, 1H), 4.02 (d, *J* = 13.6 Hz, 2H), 3.75–3.57 (m, 1H), 3.21–3.02 (m, 2H), 2.41 (s, 3H), 2.15–1.97 (m, 2H), 1.61–1.45 (m, 2H), 1.47 (s, 9H). ^13^C-NMR: 154.65, 147.22, 143.26, 130.65, 129.94, 115.06, 109.92, 79.98, 49.33, 42.11, 31.71, 28.45, 23.87.

#### 3.4.6. *tert*-Butyl 4-[(2-amino-4-bromo-5-methylphenyl)amino]piperidine-1-carboxylate (**6b**)

Hydrazine hydrate (0.94 mL, 0.97 g, 18.83 mmol, 3 eq) was slowly added to a suspension of Raney nickel (6 mL; 50% suspension in H_2_O) in a solution of **5b** (2.6 g, 6.28 mmol, 1 eq) in ethanol (30 mL). The mixture was stirred at 45 °C for 2 h and filtered over Celite. After removal of volatiles under reduced pressure and addition of saturated NaHCO_3_ (30 mL), the resulting emulsion was extracted with CH_2_Cl_2_ (3 × 30 mL). The combined organic layers were dried and concentrated under reduced pressure. The residue was purified by column chromatography (EtOAc/hexane = 1:1) followed by crystallization from hexane to afford the title compound. Yield: 0.71 g, 1.85 mmol (29%). Appearance: orange solid. ^1^H-NMR: 6.91 (s, 1H), 6.52 (s, 1H), 4.05 (d, *J* = 12.1 Hz, 2H), 3.36 (td, *J* = 9.9, 4.9 Hz, 1H), 3.25 (s, 2H), 2.97 (t, *J* = 11.4 Hz, 2H), 2.30 (s, 3H), 2.03 (d, *J* = 10.5 Hz, 2H), 1.49 (s, 9H), 1.41 (dd, *J* = 17.3, 7.3 Hz, 2H), 1.30 (dd, *J* = 12.3, 3.7 Hz, 1H); NH_2_-Group is unobservable. ^13^C-NMR: 135.57, 133.82, 129.18, 120.47, 115.20, 79.61, 50.15, 42.41, 31.71, 28.44, 22.30. MS: *m/z*: [M + H]^+^ calcd: 384.1; found: 384.1.

#### 3.4.7. *tert*-Butyl 4-(5-bromo-6-methyl-2-oxo-2,3-dihydro-1*H*-benz[d]imidazol-1-yl)-piperidine-1-carboxylate (**7**)

Et_3_N (1.15 mL, 0.83 g, 8.25 mmol, 2.01 eq) was added dropwise to a solution of **6b** (1.58 g, 4.1 mmol, 1 eq) and triphosgene (0.41 g, 1.37 mmol, 0.33 eq) in THF (10 mL). The reaction mixture was stirred at 45 °C for 2 h. After removal of volatiles under reduced pressure and addition of saturated NaHCO_3_ (30 mL), the resulting emulsion was extracted with EtOAc (3 × 30 mL). The combined organic fraction was dried and concentrated under reduced pressure. The residue was purified by column chromatography (EtOAc/hexane = 1:1) to afford the known title compound **7** [20], which was directly used for the next step. Yield: 1.59 g, 3.87 mmol (94%). Appearance: white solid. MS: *m/z*: [2M + H]^+2^ calcd: 409.6; found: 410.1.

#### 3.4.8. Deprotection of **7**

##### Procedure A

An excess of 4 m HCl in EtOAc was added to a solution of **7** (0.27 g, 0.66 mmol) in CH_2_Cl_2_ (2 mL) and the resulting mixture was stirred at ambient temperature for 1 h. After removal of volatiles under reduced pressure and addition of 0.1 m NaOH (50 mL), the resulting mixture was extracted with CH_2_Cl_2_ (3 × 30 mL). The combined organic fraction was dried and concentrated under reduced pressure to afford the crude amine hydrochloride, which was used for the next step without any purification and characterization. Yield: 0.18 g, 0.6 mmol (90% crude). Appearance: white solid.

##### Procedure B

TFA (30 mL) was slowly added to a solution of **7** (1.55 g, 3.78 mmol) in CH_2_Cl_2_ (30 mL) and the reaction mixture was stirred at ambient temperature for 1 h. After concentration under reduced pressure and addition of saturated NaHCO_3_ (30 mL) the resulting mixture was extracted with CH_2_Cl_2_ (3 × 30 mL). The combined organic fraction was dried and concentrated under reduced pressure to afford the crude amine trifluoroacetate, which was used for the next step without any purification and characterization. Yield: 1.44 g, ≤3.78 mmol (100% crude). Appearance: white solid.

#### 3.4.9. 5-Bromo-6-methyl-1-[1-(tetrahydro-2*H*-pyran-4-yl)piperidin-4-yl]-1,3-dihydro-2*H*-benz[d]imidazol-2-one (**8**). Protocol A

NaBH_3_CN (1.54 g, 24.5 mmol, 7 eq) was added to a solution of **7** (1.08 g, 3.5 mmol, 1 eq), tetrahydro-4*H*-pyran-4-one (2.45 g, 24.5 mmol, 7 eq), Bu_4_NBr (0.79 g, 2.45 mmol, 0.7 eq) and Et_3_N (1.46 mL, 1.06 g, 10.5 mmol, 3 eq) in CH_2_Cl_2_ (80 mL) and the reaction mixture was stirred for 24 h. After concentration under reduced pressure and addition of 0.1 n NaOH (50 mL), the resulting emulsion was extracted with CH_2_Cl_2_ (3 × 30 mL). The combined organic fraction was dried and concentrated under reduced pressure. The residue was purified by column chromatography (CH_2_Cl_2_/MeOH = 9:1) to afford the title compound. Yield: 83 mg, 0.21 mmol (6%). Appearance: white solid. ^1^H-NMR [(CD_3_)_2_SO + TFA; mixture of **8** and **8**·TFA]: 7.22 (s, 1H), 7.16 (s, 1H), 4.57–4.51 (m, 1H), 4.00–3.78 (m, 2H), 3.63–3.61 (m, 2H), 3.61–3.51 (m, 1H), 3.49–3.38 (m, 2H), 3.25–3.19 (m, 2H), 2.72–2.63 (m, 2H), 2.36 (s, 3H), 1.98 (m, 4H), 1.76–1.171 (m, 2H). ^13^C-NMR [(CD_3_)_2_SO + TFA; mixture of **8** and **8**·TFA]: 154.05, 153.93; 129.28, 129.24; 128.99, 128.41; 128.26, 115.39, 112.50, 110.86, 65.99, 62.08, 48.34, 47.54, 27.41, 26.26, 22.88; TFA: 158.86 (q, *J* = 37.4 Hz, CO_2_H), 115.76 (q, *J* = 289.9 Hz, CF_3_) 120.07, 117.20, 114.32, 112.50, MS: *m/z*: [2 M + MeOH + H]^+2^ calcd: 396.6; found: 396.1.

#### 3.4.10. *tert*-Butyl [1-(tetrahydro-2*H*-pyran-4-yl)piperidin-4-yl]carbamate

NaBH_3_CN (0.16 g, 2.5 mmol, 1 eq) was added to a solution of 4-*N*-Boc-aminopiperidine (0.5 g, 2.5 mmol, 1 eq), tetrahydropyran-4-one (0.3 g, 3 mmol, 1.2 eq), acetic acid (0.23 mL, 0.24 g, 4 mmol, 1.6 eq), and Bu_4_NBr (97 mg, 0.3 mmol, 0.12 eq) in CH_2_Cl_2_ (20 mL) and the reaction mixture was stirred for 2 days. Thereafter, the mixture was washed with saturated K_2_CO_3_ (10 mL), the organic layer was dried and concentrated under reduced pressure. The residue was purified by column chromatography (CH_2_Cl_2_/MeOH = 9:1) to afford the title compound. Yield: 0.34 g, 1.44 mmol (57%). Appearance: white solid. ^1^H-NMR: 4.47 (s, 1H), 4.02 (d, *J* = 10.7 Hz, 2H), 3.44 (s, 1H), 3.37 (dd, *J* = 11.7, 9.7 Hz, 2H), 2.95 (d, *J* = 12.4 Hz, 2H), 2.50 (d, *J* = 11.1 Hz, 1H), 2.29 (t, *J* = 10.6 Hz, 2H), 1.98 (d, *J* = 10.9 Hz, 2H), 1.79 (d, *J* = 9.9 Hz, 2H), 1.71–1.57 (m, 2H), 1.55 (d, *J* = 7.1 Hz, 2H), 1.45 (s, 9H). ^13^C-NMR: 144.39, 67.49, 61.23, 48.03, 32.57, 29.30, 28.44. MS: *m*/*z*: [M + H]^+^ calcd: 285.2 found: 285.2.

#### 3.4.11. 1-(Tetrahydro-2*H*-pyran-4-yl)piperidine-4-amine (**10**)

A solution of anhydrous HCl in EtOAc was prepared by addition of AcCl (2.25 mL, 2.48 g, 31.6 mmol, 4.9 eq) to an ice-cold solution of MeOH (1.34 mL, 1.06 g, 33 mmol, 5.2 eq) in EtOAc (8.25 mL). The resulting solution was added to a solution of *tert*-butyl [1-(tetrahydro-2*H*-pyran-4-yl)piperidin-4-yl]carbamate (1.83 g, 6.4 mmol, 1 eq) in CH_2_Cl_2_ (5 mL) and the mixture was stirred for 2 h. Volatiles were removed under reduced pressure, the residue was taken up in 0.1 m NaOH (50 mL) and the resulting mixture was extracted with CH_2_Cl_2_ (3 × 30 mL). The combined organic fraction was dried and concentrated under reduced pressure to afford the title compound, which was directly used for the next step. Yield: 1.1 g, 5.96 mmol (93%). Appearance: yellow solid. ^1^H-NMR (CD_3_OD + TFA; mixture of 10·TFA and 10·2 TFA): 4.10–4.04 (m, 2H), 3.75–3.72 (m, 2H), 3.59–3.41 (m, 4H), 3.25–3.19 (m, 2H), 2.36–2.34 (m, 2H), 2.19–2.05 (m, 4H), 1.91–1.81 (m, 2H). MS: *m*/*z*: [M + H]^+^ calcd: 185.2; found: 185.4.

#### 3.4.12. *N*-(4-Fluoro-5-methyl-2-nitrophenyl)-1-(tetrahydro-2*H*-pyran-4-yl)piperidine-4-amine (**11a**)

A solution of **10** (0.184 g, 1 mmol, 1 eq), **4a** (0.173 g, 1 mmol, 1 eq) and DIEA (0.175 mL, 0.13 g, 5.8 mmol, 5.8 eq) in DMF (5 mL) was stirred at 70 °C for 16 h. After removal of volatiles under reduced pressure and addition of H_2_O (30 mL), the resulting mixture was extracted with CH_2_Cl_2_ (3 × 10 mL). The combined organic fraction was dried and concentrated under reduced pressure. The residue was purified by column chromatography (CHCl_3_/acetone = 1:5) to afford the title compound which was directly used for the next step without any characterization. Yield: 0.19 g, 0.58 mmol (58%). Appearance: red-orange solid.

### 3.4.13. 4-Fluoro-5-methyl-N-(1-(tetrahydro-2H-pyran-4-yl)piperidin-4-yl)benzene-1,2-diamine

Hydrazine hydrate (56 µL, 61 mg, 0.95 mmol, 1.7 eq) was slowly added to a suspension of Raney nickel (0.5 mL 50% suspension in H_2_O) in a solution of **11a** (0.19 g, 0.56 mmol, 1 eq) in EtOH (5 mL). The reaction mixture was stirred at 45 °C for 2 h and filtered over Celite. After removal of volatiles under reduced pressure saturated NaHCO_3_ (20 mL) was added and the resulting mixture was extracted with CH_2_Cl_2_ (3 × 30 mL). The combined organic fraction was dried and concentrated under reduced pressure. The residue was purified by column chromatography (MeOH/CH_2_Cl_2_ = 1:6) and recrystallized from hexane to afford the title compound which was used for the next step without any characterization. Yield: 0.11 g, 0.35 mmol (63%). Appearance: brown solid.

### 3.4.14. 5-Fluoro-6-methyl-1-[1-(tetrahydro-2H-pyran-4-yl)piperidin-4-yl]-1,3-dihydro-2H-benz[d]-imidazole-2-one (**1**)

Et_3_N (98 µL, 71 mg 0.7 mmol, 2 eq) was added dropwise to a solution of 4-fluoro-5-methyl-*N*-(1-(tetrahydro-2*H*-pyran-4-yl)piperidin-4-yl)benzene-1,2-diamine (0.1 g, 0.35 mmol, 1 eq) and triphosgene (35 mg, 0.12mmol, 0.34 eq) in THF (5 mL). The mixture was stirred at 45 °C for 2 h. After removal of volatiles under reduced pressure and addition of saturated NaHCO_3_ (10 mL), the mixture was extracted with CH_2_Cl_2_ (3 × 10 mL). The combined organic fraction was dried and concentrated under reduced pressure. The residue was purified by column chromatography (MeOH/CH_2_Cl_2_ = 1:6) and recrystallized from CH_2_Cl_2_/hexane to afford **1** [9]. Yield: 70 mg, 0.2 mmol (60%). Appearance: white solid. ^1^H-NMR: 9.90 (s, 1H), 7.07 (d, *J* = 4.6 Hz, 1H), 6.83 (d, *J* = 9.1 Hz, 1H), 4.35 (s, 1H), 4.08 (d, *J* = 9.2 Hz, 2H), 3.43 (t, *J* = 11.4 Hz, 2H), 3.15 (d, *J* = 4.1 Hz, 2H), 2.69–2.51 (m, 1H), 2.50–2.21 (m, 4H), 2.31 (s, 3H), 1.98–1.49 (m, 6H). ^19^F-NMR: −124.79 (s).

### 3.4.15. *N*-(4-Bromo-5-methyl-2-nitrophenyl)-1-(tetrahydro-2*H*-pyran-4-yl)piperidine-4-amine (**11b**)

A solution of **10** (1.07 g, 5.8 mmol, 1 eq), **4b** (1.36 g, 5.8 mmol, 1 eq) and DIEA (1 mL, 0.74 g, 5.8 mmol, 1 eq) in DMF (15 mL) was stirred at 70 °C for 16 h. After removal of volatiles under reduced pressure, the residue was taken up with H_2_O (50 mL), and the mixture was extracted with CH_2_Cl_2_ (3 × 10 mL). The combined organic fraction was dried and concentrated under reduced pressure affording the title compound (1.5 g, 66%). ^1^H-NMR (CDCl_3_): 8.35 (s, 1H),8.10 (t, *J* = 10.6 Hz, 1H), 6.74 (s, 1H), 4.07–4.04 (m, 2H), 3.57–3.55 (m, 1H), 3.43–3.38 (m, 2H), 2.93–2.91 (m, 2H), 2.59–2.45 (m, 3H), 2.41 (s, 3H), 2.19–2.05 (m, 2H), 1.79–1.68 (m, 6H). ^13^C-NMR (CDCl_3_): 147.06, 143.5, 130.60, 129.85, 115.13, 109.62, 67.54, 61.08, 49.12, 47.17, 31.93, 29.38, 23.82. MS: *m*/*z*: [M + H]^+^ calcd: 398.1; found: 398.1.

### 3.4.16. 4-Bromo-5-methyl-*N*-(1-(tetrahydro-2*H*-pyran-4-yl)piperidin-4-yl)benzene-1,2-diamine

Zn powder (2.66 g, 40.7 mmol, 10.7 eq) was added to a stirred solution of crude **11b** (1.5 g, max. 3.8 mmol, 1 eq) and NH_4_Cl (2.18 g, 3.8 mmol, 1 eq) in a mixture of EtOAc/EtOH 1:1 (50 mL). The reaction mixture was stirred overnight, then diluted with EtOAc (100 mL) and filtered over Celite. The filtrate was concentrated under reduced pressure. The residue was purified by column chromatography (MeOH/CH_2_Cl_2_, 1:6) followed by recrystallization from hexane to afford the title compound. Yield: 1.26 g, 3.42 mmol (58% over two steps). Appearance: brown solid. ^1^H-NMR: 6.90 (s, 1H), 6.51 (s, 1H), 4.05 (d, *J* = 8.7 Hz, 2H), 3.40 (t, *J* = 10.9 Hz, 2H), 3.25 (s, 2H), 2.96 (d, *J* = 11.6 Hz, 2H), 2.52 (s, 1H), 2.45–2.32 (m, 2H), 2.29 (s, 3H), 2.09 (d, *J* = 12.0 Hz, 2H), 1.81 (s, 1H), 1.76 (s, 2H), 1.69–1.34 (m, 4H). ^13^C-NMR: 135.96, 133.65, 129.18, 120.37, 119.87, 115.00, 67.65, 61.12, 50.22, 47.94, 32.83, 29.50, 22.39. MS: *m/z*: [M + H]^+^ calcd: 368.1; found: 368.1.

### 3.4.17. 5-Bromo-6-methyl-1-[1-(tetrahydro-2*H*-pyran-4-yl)piperidin-4-yl]-1,3-dihydro-2*H*-benz[d]imidazol-2-one (**8**). Protocol B

Et_3_N (0.75 µL, 0.55 g, 5.4 mmol, 2 eq) was added dropwise to a solution of 4-bromo-5-methyl-*N*-(1-(tetrahydro-2*H*-pyran-4-yl)piperidin-4-yl)benzene-1,2-diamine (1 g, 2.7 mmol, 1 eq) and triphosgene (0.27 g, 0.9 mmol, 0.33 eq) in THF (60 mL). The mixture was stirred at 45 °C for 2 h. After removal of volatiles under reduced pressure and addition of saturated NaHCO_3_ (60 mL), the resulting mixture was extracted with CH_2_Cl_2_ (3 × 60 mL). The combined organic fraction was dried and concentrated under reduced pressure to 5–7 mL. Hexane (50–70 mL) was added and the precipitated solid was filtered off furnishing the title compound, which was directly used for the next step. Yield: 0.56 g, 1.40 mmol (52%).

### 3.4.18. 6-Methyl-1-[1-(tetrahydro-2*H*-pyran-4-yl)piperidin-4-yl]-5-(4,4,5,5-tetramethyl-1,3,2-dioxaborolan-2-yl)-1,3-dihydro-2*H*-benz[d]imidazol-2-one (**2**)

A suspension KOAc (0.39 g, 3.9 mmol, 3 eq) in a solution of **8** (0.51 g, 1.3 mmol, 1 eq), Pd(dppf)Cl_2_ (47.5 mg, 65 µmol, 0.05 eq) and bis(pinacolato)diboron (0.41 g, 1.63 mmol, 1.25 eq) in anhydrous dioxane (6 mL) was stirred at 110 °C for 3 h. After removal of the volatiles under reduced pressure and addition of 0.1 n NaOH (5 mL), the mixture was extracted with CH_2_Cl_2_ (3 × 20 mL). The combined organic fraction was dried and concentrated under reduced pressure. The residue was purified by column chromatography (MeOH/CH_2_Cl_2_ = 1:10) and recrystallized from CH_2_Cl_2_/hexane to afford the title compound. Yield: 0.28 g, 0.62 mmol (48%). Appearance: white solid. ^1^H-NMR: 10.04 (s, 1H), 7.53 (d, *J* = 19.9 Hz, 1H), 7.12 (s, 1H), 4.37 (s, 1H), 4.09 (d, *J* = 9.5 Hz, 2H), 3.43 (t, *J* = 11.4 Hz, 2H), 3.16 (s, 2H), 2.60 (s, 3H), 2.69–2.25 (m, 2H), 2.05–1.55 (m, 6H), 1.33 (s, 12H). ^13^C-NMR: 155.44, 125.42, 116.74, 113.08, 111.02, 109.18, 83.35, 67.63, 48.83, 29.70, 24.89, 22.35; C-Bpin was not observed. MS: *m*/*z*: [M + H]^+^ calcd: 442.3; found: 442.3.

### 3.5. Radiochemistry

#### 3.5.1. General

[^18^F]Fluoride was produced by the ^18^O (p,n) ^18^F reaction by bombardment of enriched [^18^O] water with 17 MeV protons at the BC1710 cyclotron (The Japan Steel Works, Tokyo, Japan) of the INM-5 (Forschungszentrum Jülich).

Radioactivity was measured using a CRC-55tR Dose Calibrator from Capintec, Inc. (Florham Park, Netherlands).

All radiosyntheses were carried out in 5 mL V-Vials (Wheaton) equipped with PTFE wing coated stir bars using anhydrous DMA (Aldrich), anhydrous *n*BuOH and anhydrous MeOH dried over molecular sieves (both Acros Organics, Geel, Belgium). Cu(OTf)_2_(py)_4_ was prepared according to the literature [21] and stored under ambient conditions without any precautions.

Radiolabeling experiments were performed using AREX-9 Digital Pro hot plate equipped with VTF digital thermometer (VELP SCIENTIFICA, Usmate Velate MB, Italy), vacuum pump Laboport (KNF, Freiburg im Breisgau, Germany) in the customized hot cell installed by Von Gahlen Nederland B.V (Zevenaar, Netherlands).

Radiosyntheses were carried out under synthetic air (80% N_2_ + 20% O_2_) or Ar.

Sep-Pak Plus C18 cartridges and Sep-Pak Accell Plus QMA carbonate plus light cartridges, 46 mg sorbent per cartridge, (both Waters GmbH, Eschborn, Germany) were applied.

HPLC analyses and preparative separations were carried out using an Ultimate® 3000 HPLC system from Thermo Fisher Scientific (Sunnyvale, CA, USA) with variable wavelength detector coupled in series with a HERM LB 500 radio-flow monitor (Berthold Technologies, Bad Wildbad, Germany). The unselective adsorption of ^18^F^−^ onto HPLC columns was determined to be <10% in each case. The UV and radioactivity detectors were connected in sequence, giving a time delay of 0.1–0.9 min between the corresponding responses, depending on the flow rate. The identity of [^18^F]**1** was confirmed by the co-injection of the non-radioactive reference compound.

Analytical HPLC

Column: Luna C18 (2), 250 × 4.6 mm (Phenomenex, Aschaffenburg, Germany); eluent: 20% MeCN (0.1% TFA); flow rate: 1.5 mL/min.

Preparative HPLC

Column: Luna C18 (2), 250 × 10 mm (Phenomenex, Aschaffenburg, Germany); eluent: 25% MeCN (0.1% TFA); flow rate: 4 mL/min.

#### 3.5.2. Processing [^18^F]fluoride

Aqueous [^18^F]fluoride (0.02–7 GBq) was loaded onto an anion-exchange resin (QMA cartridge) from the male side. The resin was flushed with MeOH (2 mL) and dried with 5–10 mL of air from the male side. ^18^F^−^ was *slowly* eluted into the reaction vial from the female side using a solution of Et_4_NHCO_3_ (2 mg, 10 μmol) in MeOH (0.5 mL). It should be noted that flushing with MeOH was carried out from the male side whereas drying and ^18^F^−^ elution were carried out from the female side. If the QMA cartridge had been loaded, flushed, and eluted from the female side only, sometimes a significant amount of [^18^F]fluoride remained on the resin, probably because QMA light cartridges have a single frit on the male side but four frits on the female side. MeOH was evaporated using a flow of air at 80 °C within 10 min. Using this procedure, recovery of ^18^F^−^ amounted to ≥85%.

#### 3.5.3. 5-[^18^F]Fluoro-6-methyl-1-(1-(tetrahydro-2*H*-pyran-4-yl)piperidin-4-yl)-1,3-dihydro-2*H*-benz-[d]-imidazol-2-one ([^18^F]**1**)

A solution of the pinacol boronate precursor **2** (7.2 mg, 16 μmol, 1 eq) and Cu(py)_4_(OTf)_2_ (13.6 mg, 20 μmol, 1.25 eq) in DMA/*n*BuOH 2:1 (750 μL) was added to [^18^F]Et_4_NF, and the resulting solution was heated at 110 °C for 10 min under air or argon. For the determination of RCC, the reaction mixture was cooled to <40 °C, 0.1% TFA (1 mL) was added and the mixture was stirred for 30 s. HPLC analysis demonstrated that [^18^F]**1** formed in a RCC of 96 ± 3% (*n* = 3). For isolation of [^18^F]**1**, the reaction mixture was concentrated at 110 °C for 10 min, the residue was taken up in 25% MeCN (0.1% TFA) and the mixture was purified by preparative HPLC. The fraction containing [^18^F]**1** was diluted with a 10-fold volume of H_2_O and loaded onto a C18 cartridge. The cartridge was washed with 5% MeCN (10 mL), H_2_O (5 mL) and dried with air (10 mL). The tracer was eluted with EtOH (1 mL). EtOH was evaporated at 90 °C and the residue was taken up in saline for injection affording [^18^F]**1** as a ready-to-use solution in 13–19% n.d.c RCY (Table 1).

## 4. Conclusions

We developed a straightforward and efficient protocol for the manual preparation of [^18^F]**1**, a potentially M_1_ selective PET-probe for in vivo studies. The protocol is amenable to automation owing to its simplicity, and thus provides an important step towards the routine production of ^18^F-labeled M_1_ selective PET tracers for experimental and diagnostic applications.

## Figures and Tables

**Table 1:** Preparation of [^18^F]**1** on a preparative scale.

^18^F^−^ (MBq)	[^18^F]1 (MBq)	n.d.c. RCY (%)
3300	460	13
3970	770	19
1850	340	18

## Supplementary Materials

The following are available online at https://www.mdpi.com/1420-3049/25/12/2880/s1, ^1^H-, ^13^C- and ^19^F-NMR spectra and determination of molar activity and carrier amount.
