# Supplementary material for: Preparation of a First 18F-Labeled Agonist for M1 Muscarinic Acetylcholine Receptors
Source: Molecules. 2020 Jun 23;25(12):2880. doi: 10.3390/molecules25122880 (PMC7355535; doi:10.3390/molecules25122880)
Supplement: Supplementary file 1 [file molecules-25-02880-s001.pdf]

Supporting Information

Preparation of a First ^{18}F-Labeled Agonist for M_1 Muscarinic Acetylcholine Receptors

Boris D. Zlatopolskiy [1,2,3], Felix Neumaier [4], Till Rüngeler [2], Birte Drewes [1], Niklas Kolks [1,2] and Bernd Neumaier [1,2,3,*]

[1] Institute of Neuroscience and Medicine, Nuclear Chemistry (INM-5), Forschungszentrum Jülich GmbH, 52428 Jülich, Germany; boris.zlatopolskiy@uk-koeln.de (B.D.Z.); b.drewes@fz-juelich.de (B.D.); niklas.kolks@uk-koeln.de (N.K.)
[2] Institute of Radiochemistry and Experimental Molecular Imaging, University Clinic Cologne, 50931 Cologne, Germany; till@ruengeler.eu
[3] Max Planck Institute for Metabolism Research, 50931 Cologne, Germany
[4] Institute of Neurophysiology, University Hospital Cologne, Robert-Koch Str. 39, 50931 Cologne, Germany; felix@neumaier-net.de
* Correspondence: b.neumaier@fz-juelich.de; Tel.: +49-2461-61-4141

Table of Content

NMR-Spectra

Determination of carrier amount and molar activity

NMR-Spectra

5-Fluoro-6-methyl-1-[1-(tetrahydro-2*H*-pyran-4-yl)piperidin-4-yl]-1,3-dihydro-2H-benz[d]-imidazole-2-one (**1**)

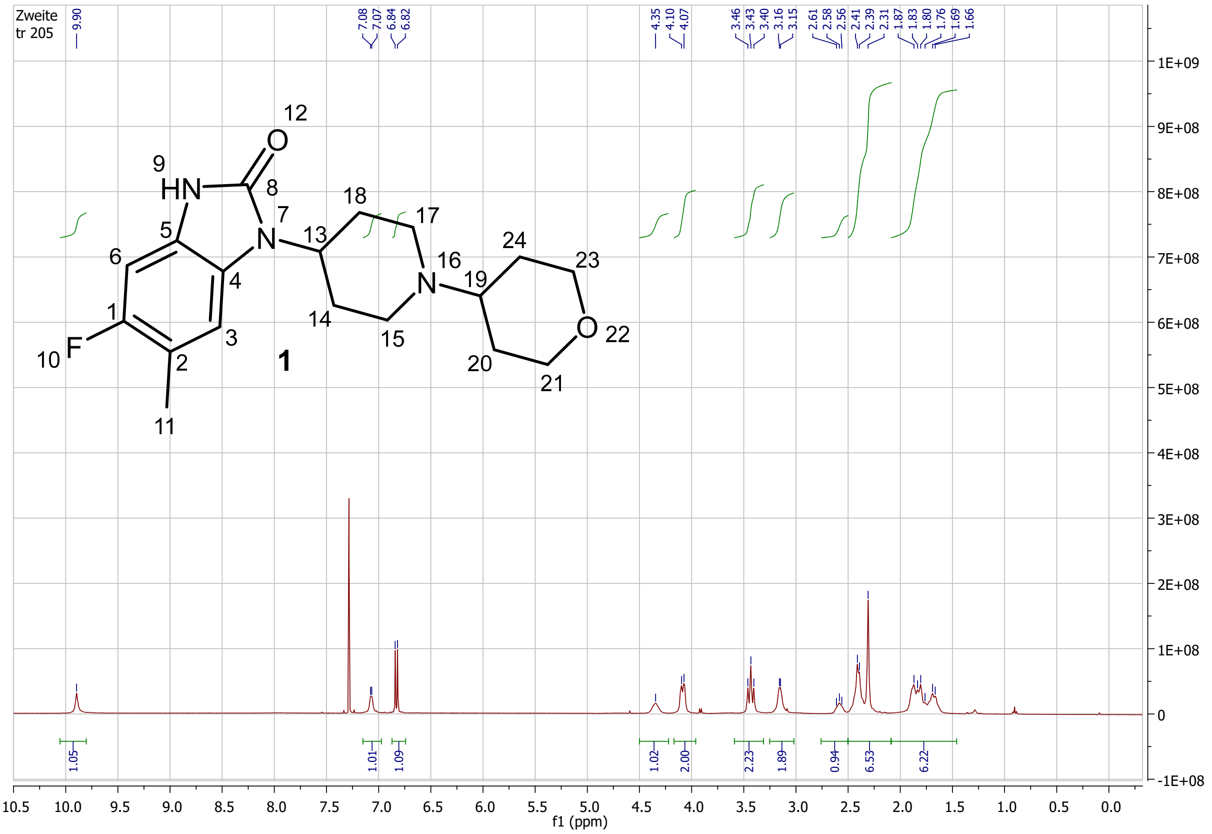

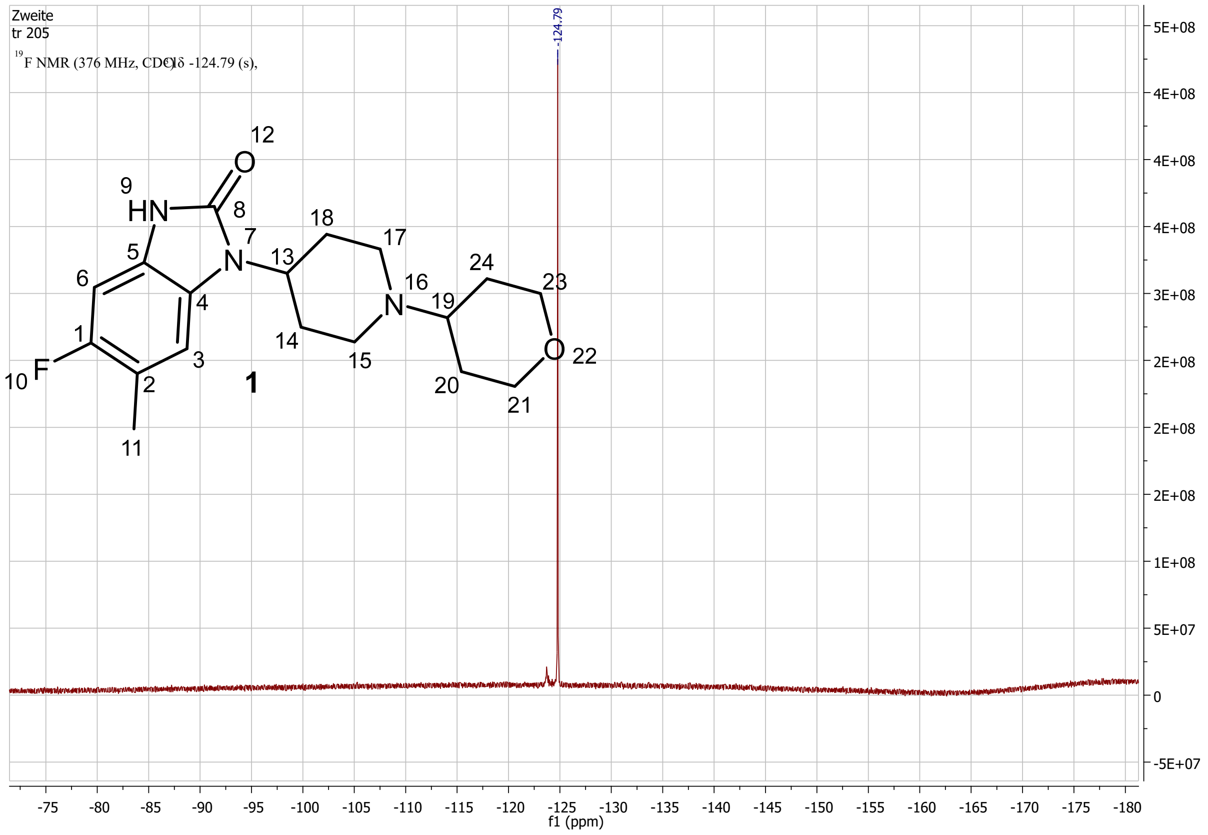

6-Methyl-1-[1-(tetrahydro-2*H*-pyran-4-yl)piperidin-4-yl]-5-(4,4,5,5-tetramethyl-1,3,2-dioxaborolan-2-yl)-1,3-dihydro-2H-benz[d]imidazol-2-one (**2**)

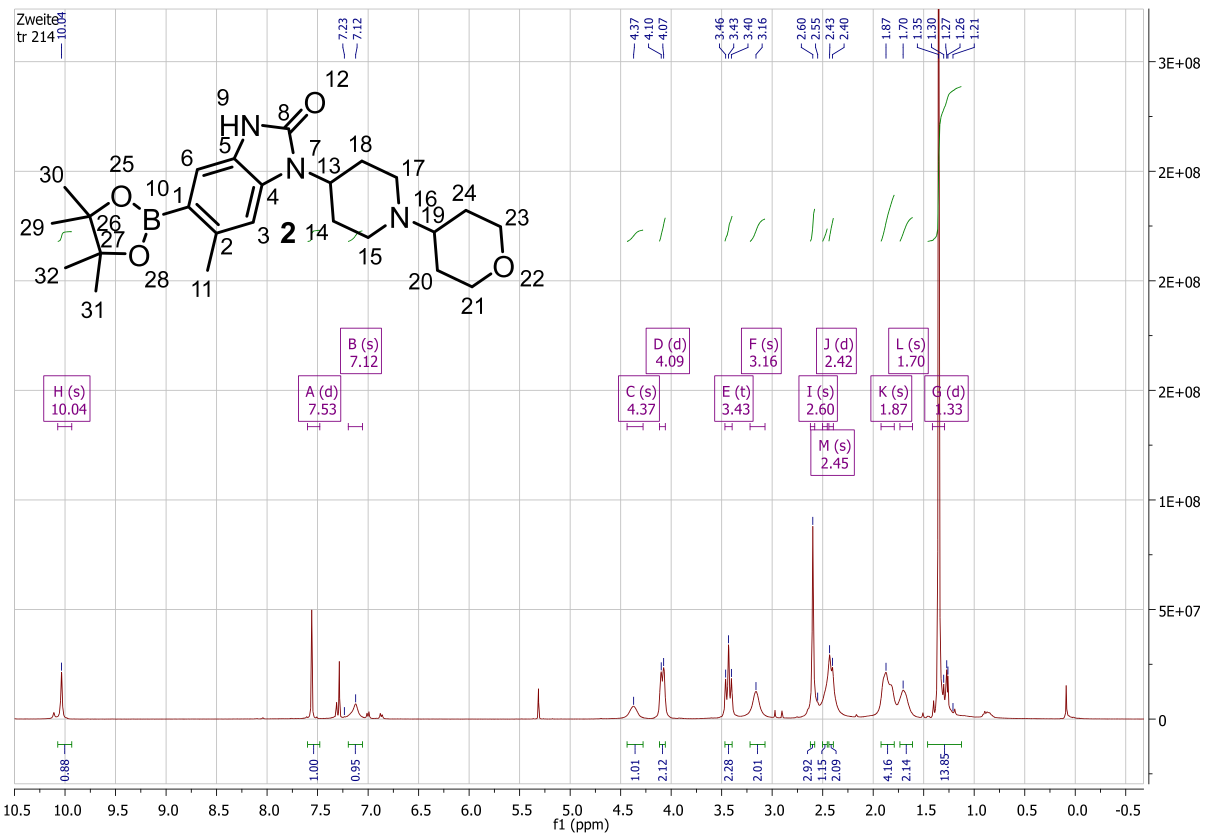

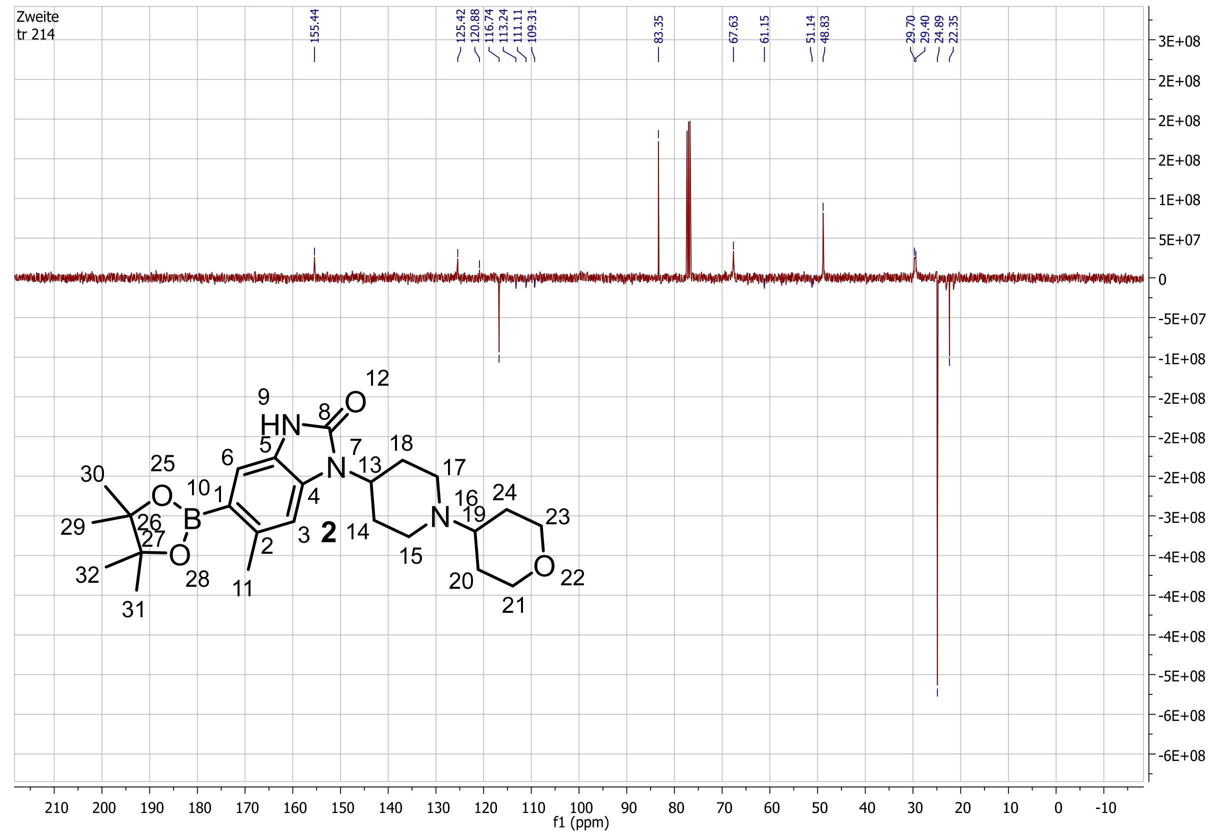

2,5-Difluoro-4-nitrotoluene (**4a**)

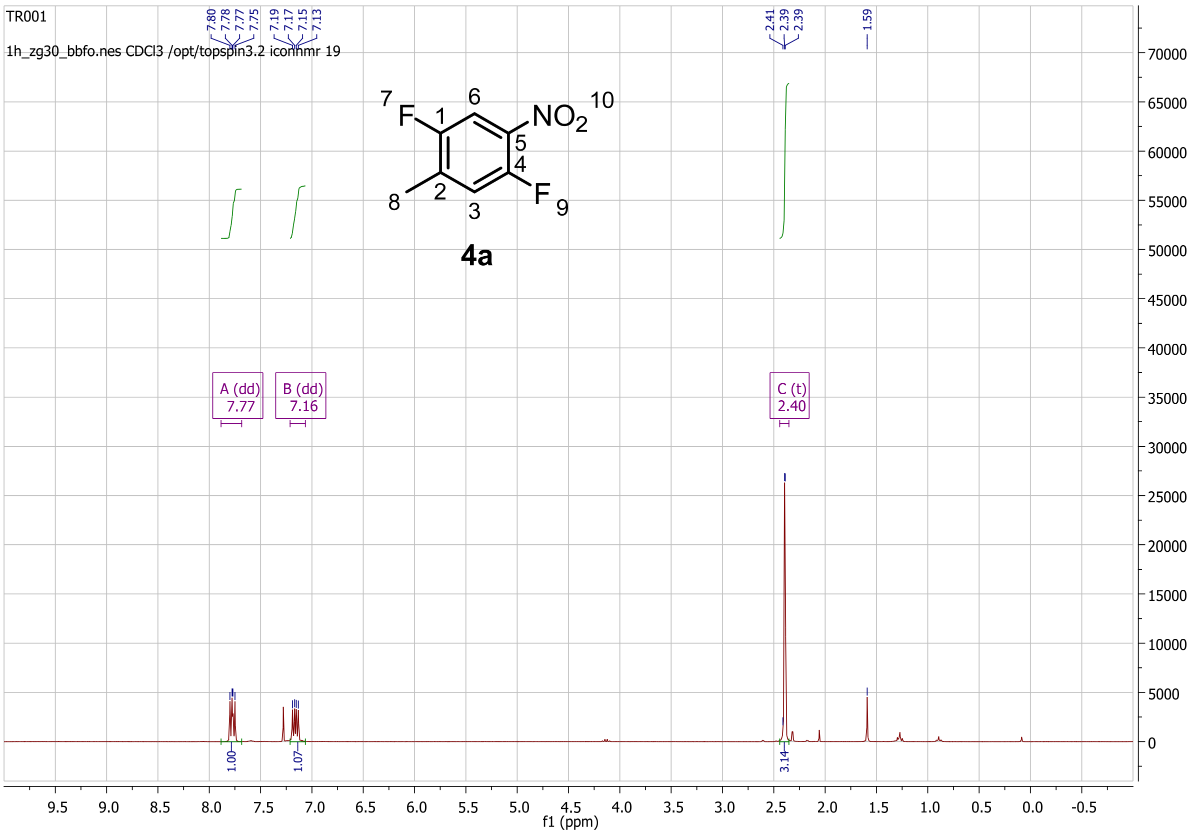

2-Bromo-5-fluoro-4-nitrotoluene (**4b**)

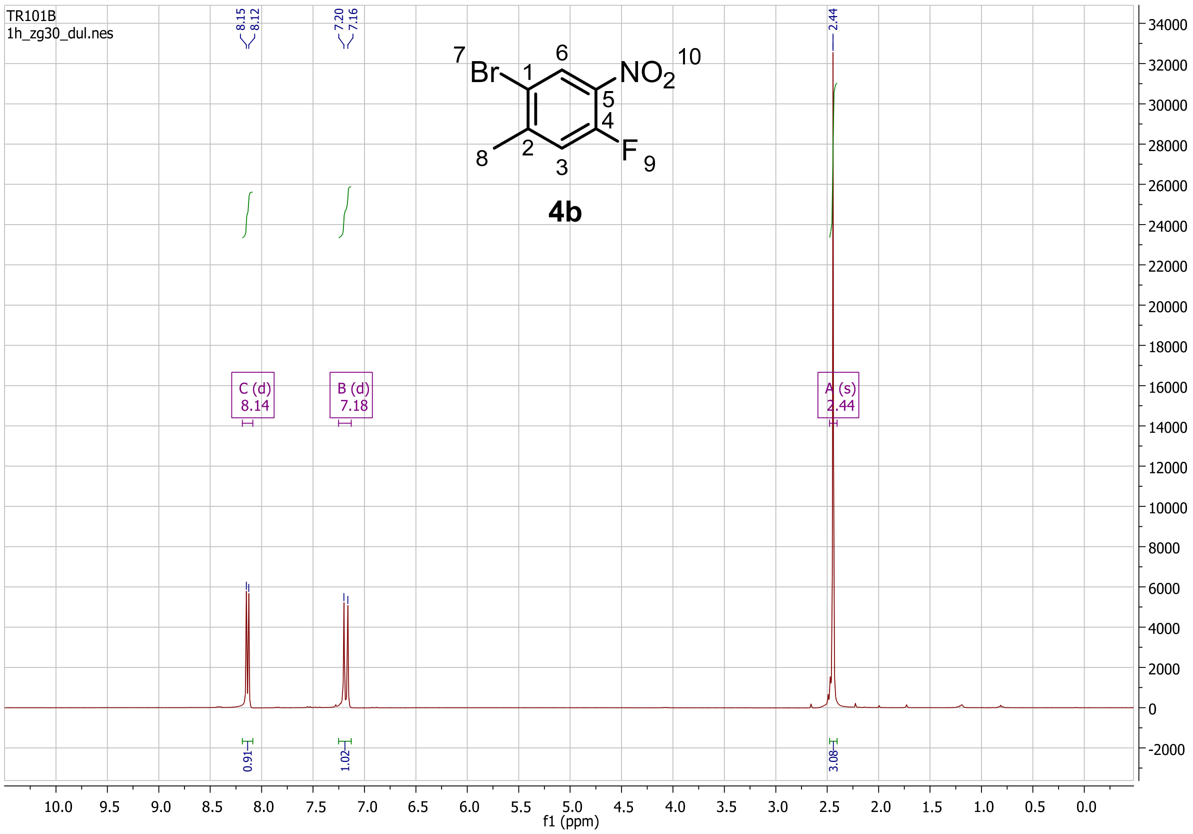

tert-Butyl 4-[(4-fluoro-5-methyl-2-nitrophenyl)amine]piperidine-1-carboxylate (**5a**)

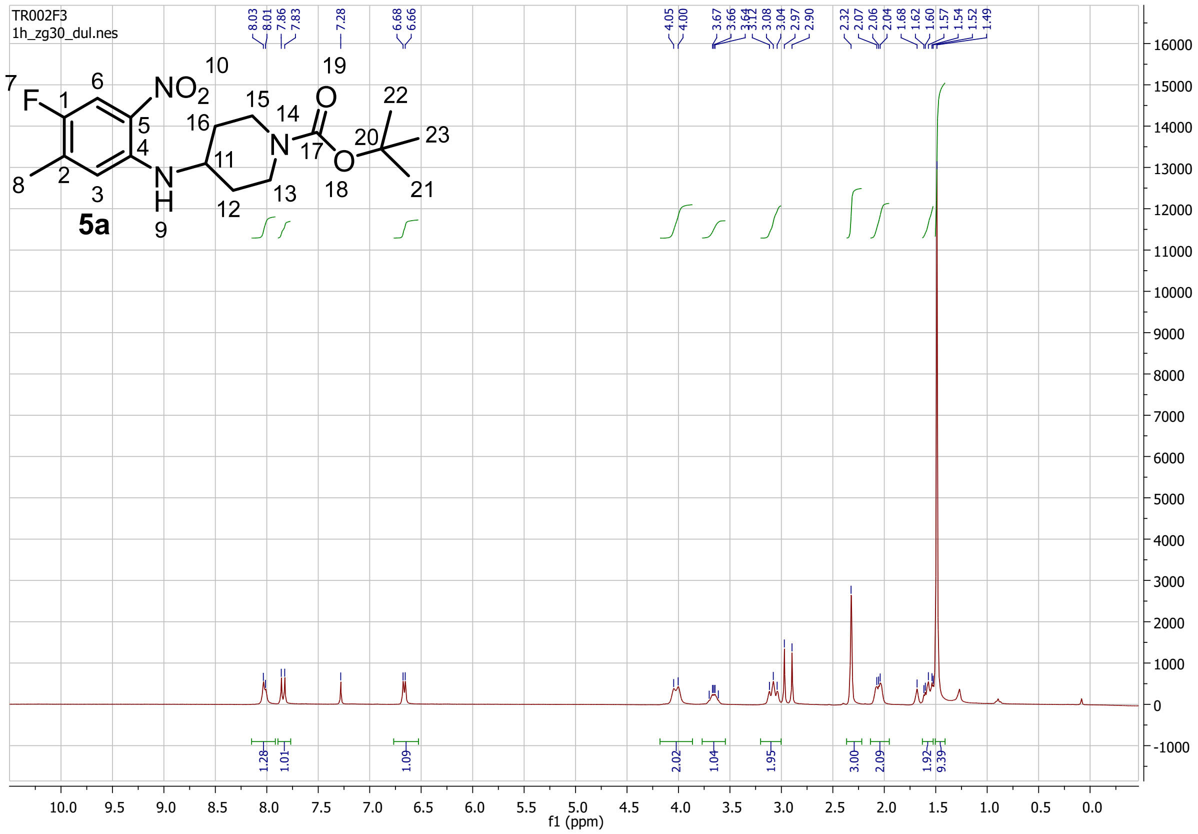

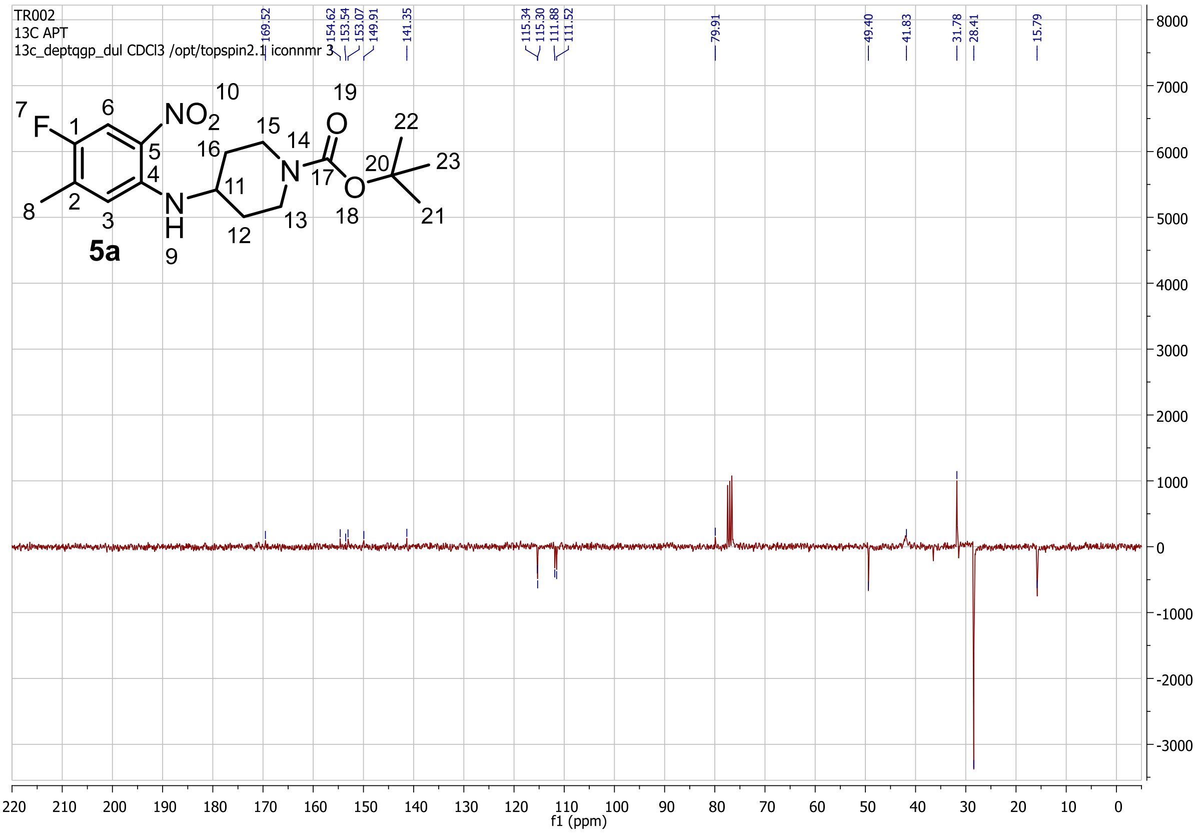

tert-Butyl 4-[(4-bromo-5-methyl-2-nitrophenyl)amine]piperidine-1-carboxylate (**5b**)

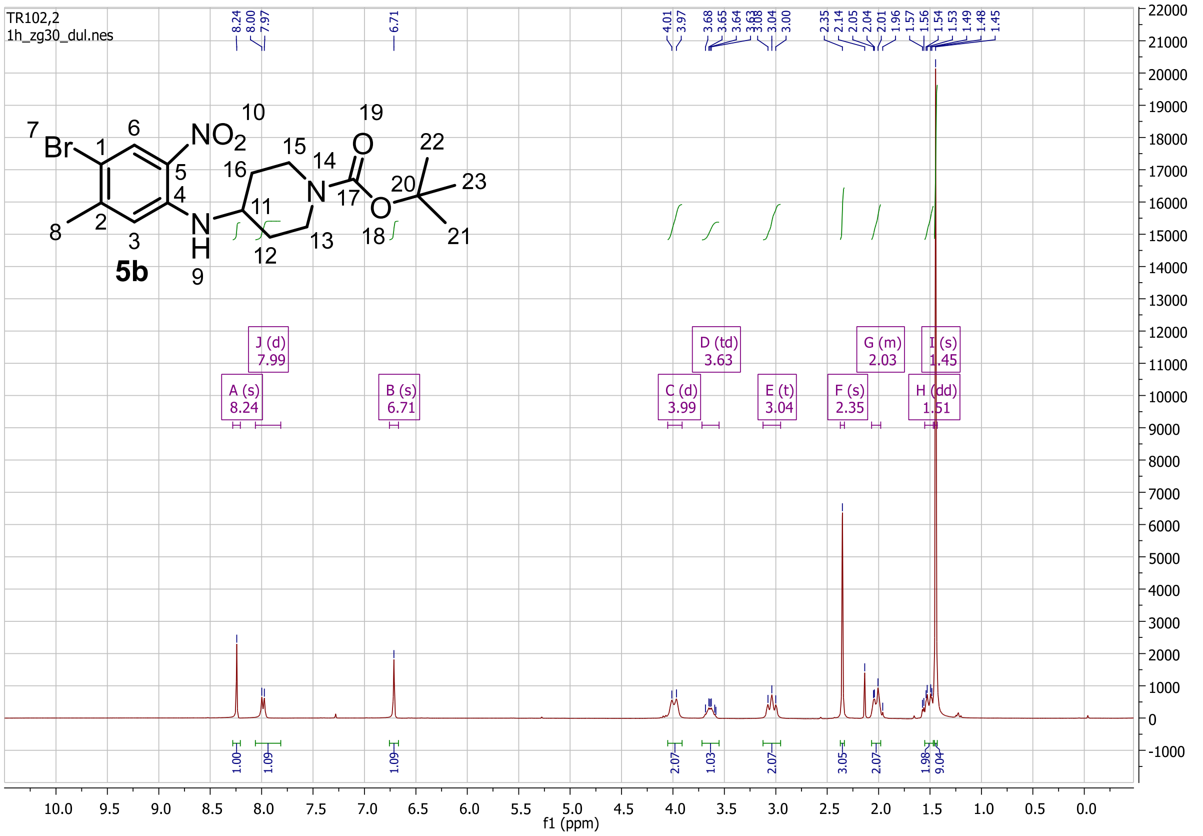

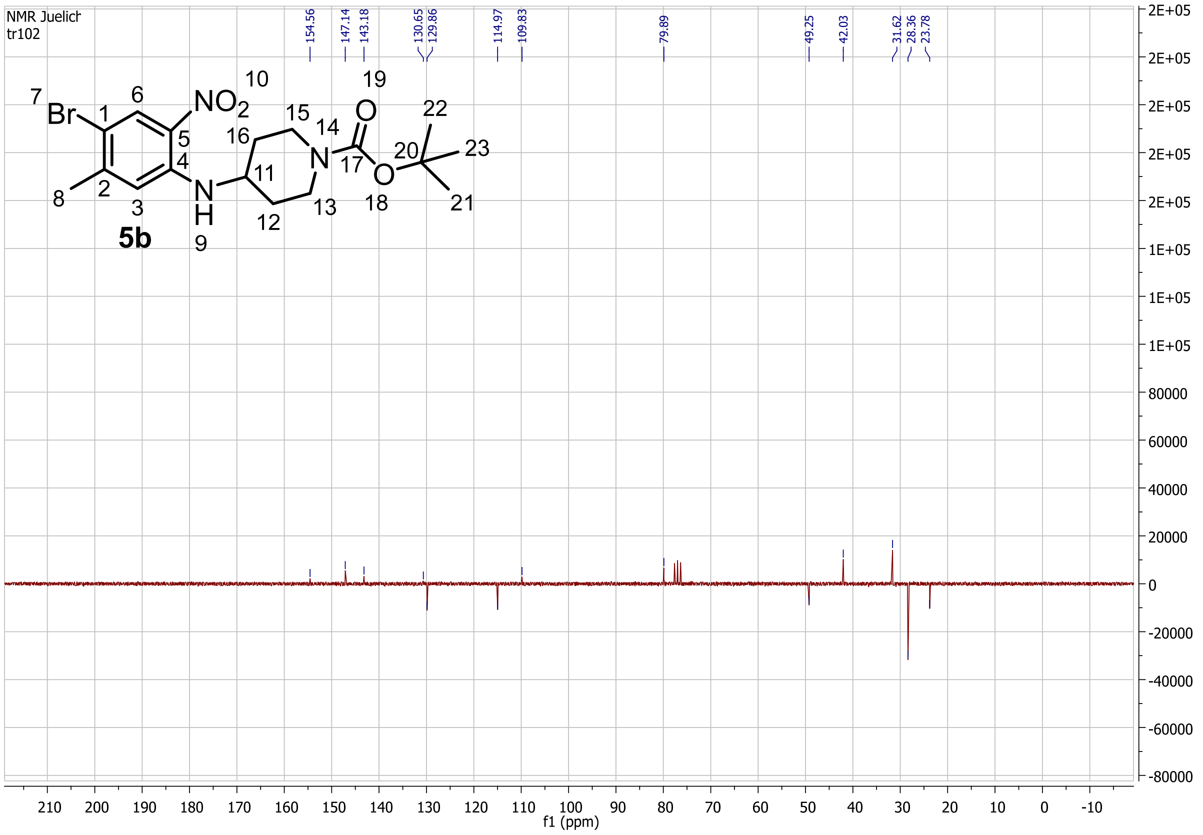

tert-Butyl 4-[(2-amine-4-bromo-5-methylpheny)]amine]piperidine-1-carboxylate (**6b**)

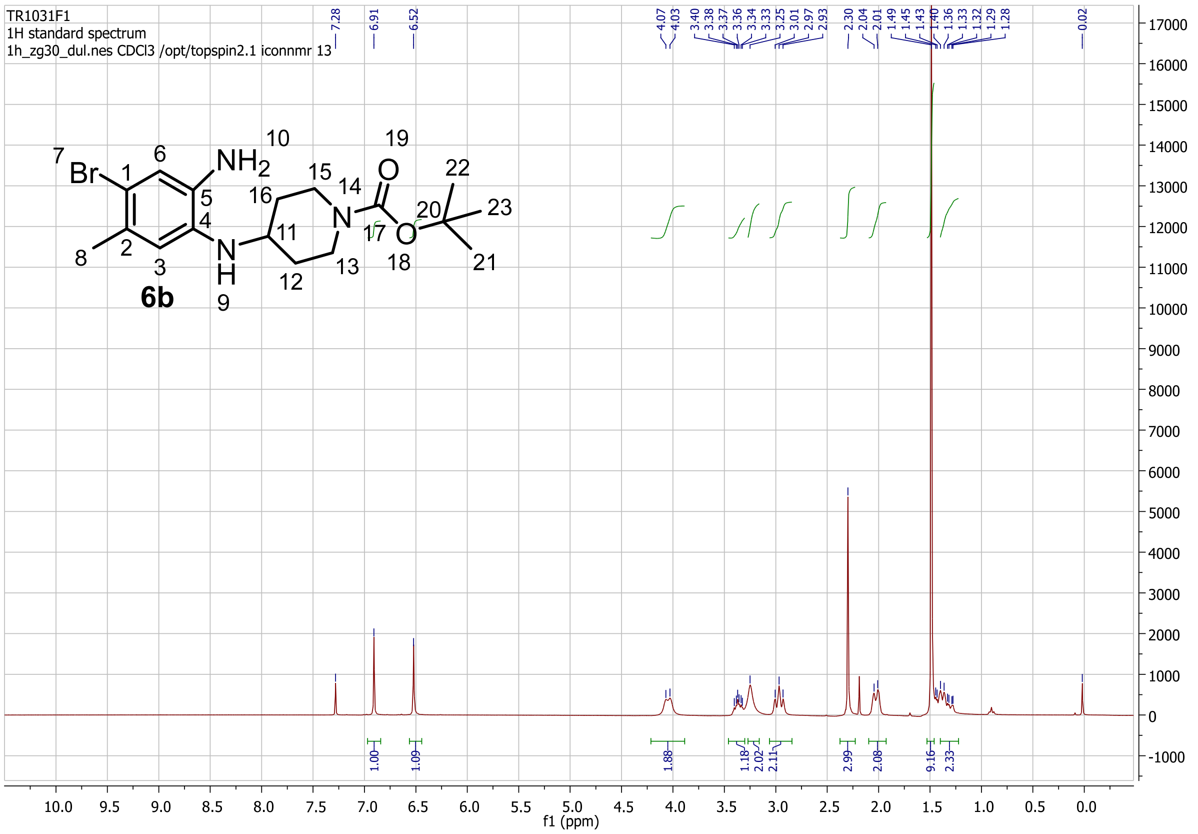

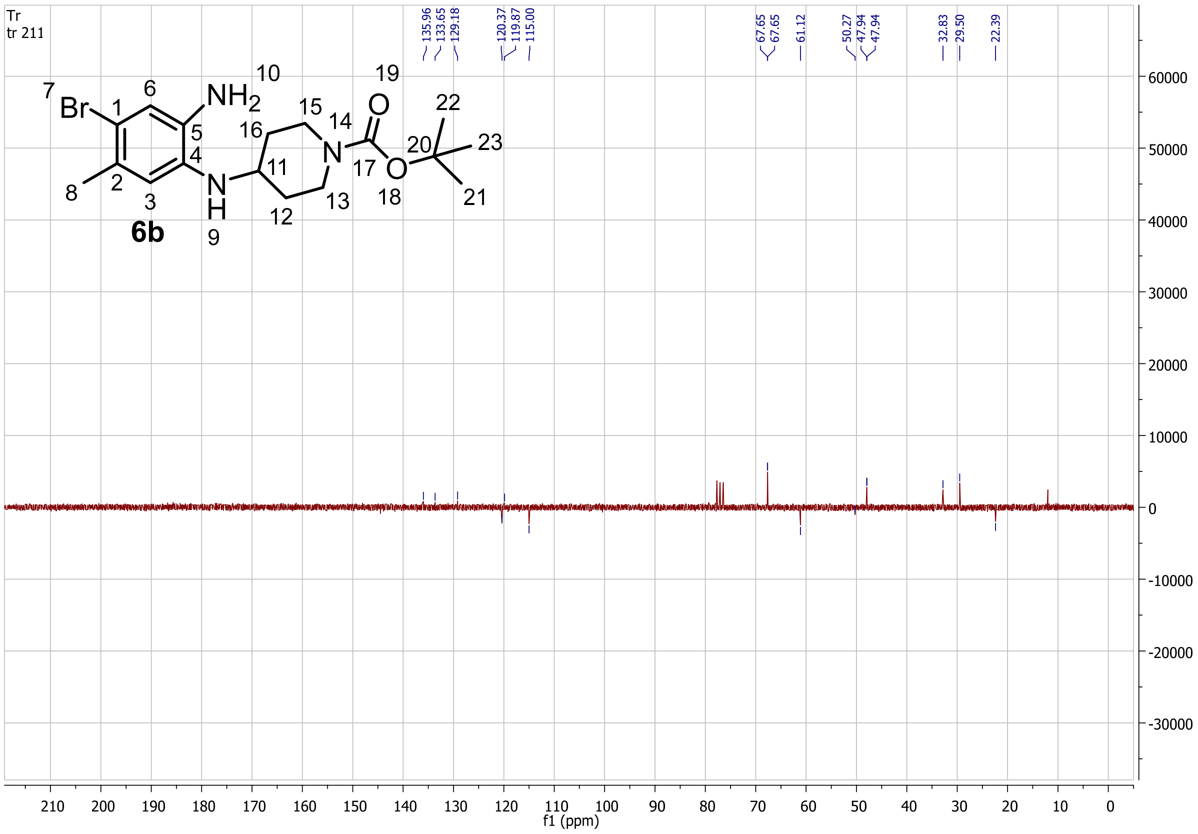

5-Bromo-6-methyl-1-[1-(tetrahydro-2*H*-pyran-4-yl)piperidin-4-yl]-1,3-dihydro-2*H*-benz[d]imidazol-2-one (mixture of **8** and **8**·TFA)

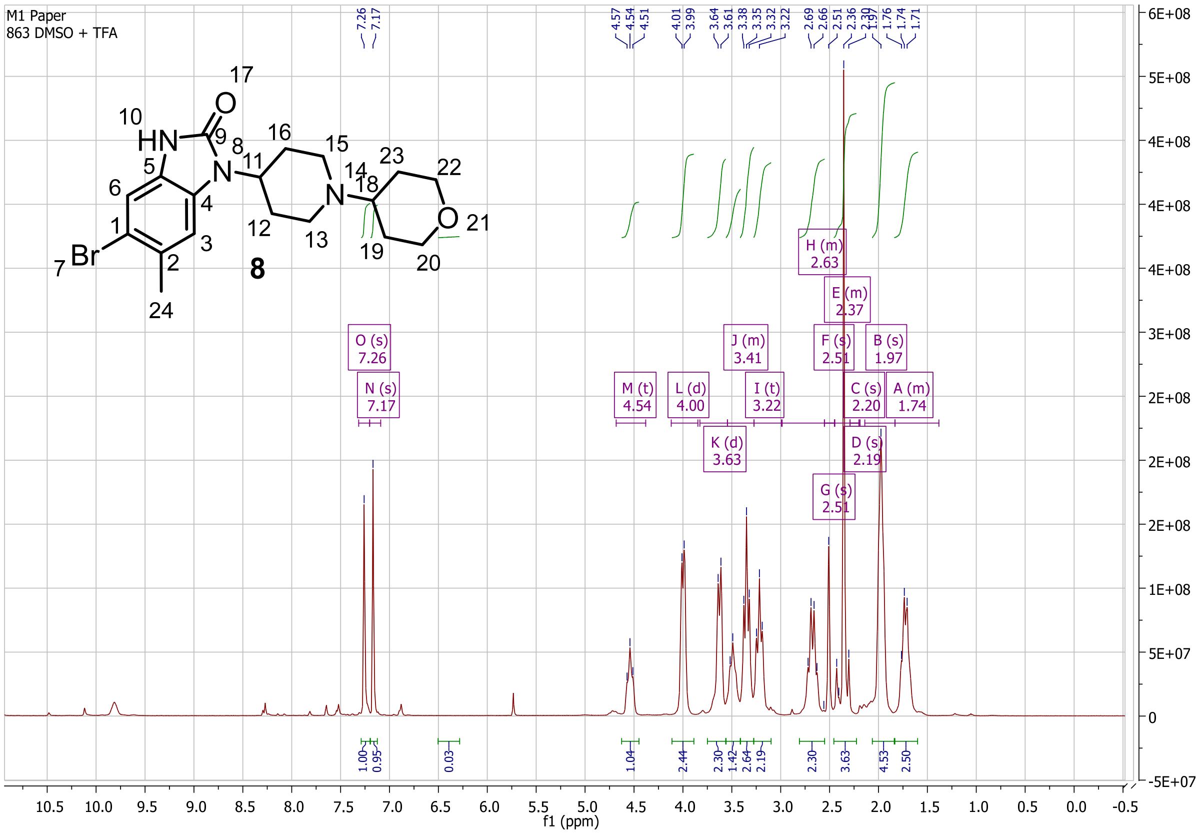

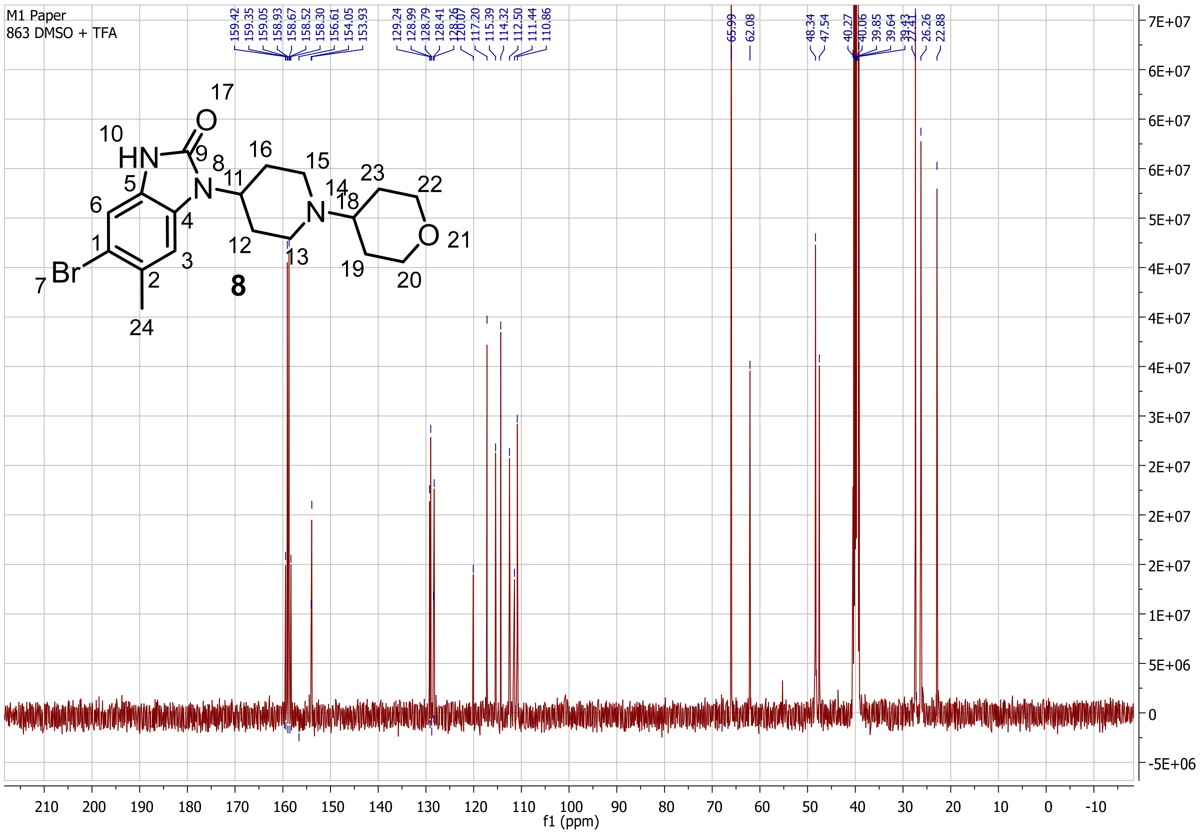

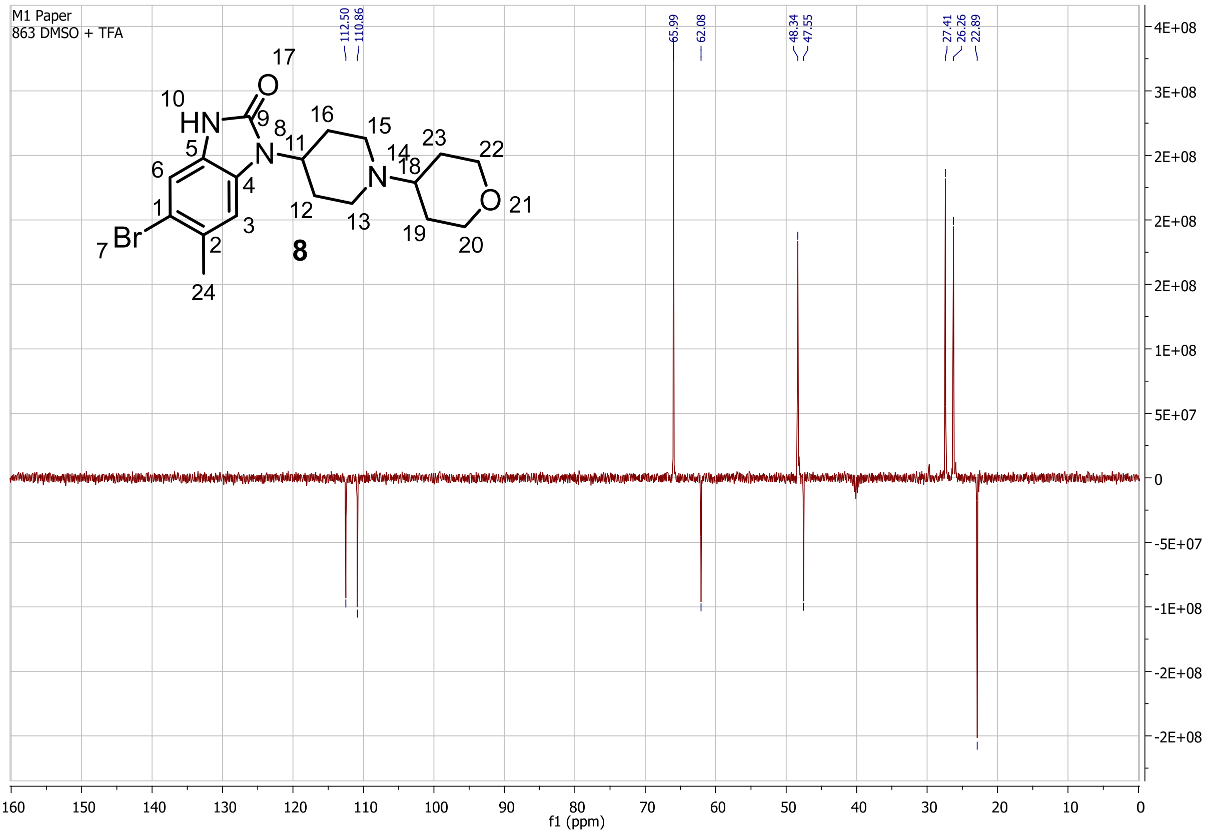

M1 Paper
863 DMSO + TFA
112.50
110.86
65.99
62.08
48.34
47.55
27.41
26.26
22.89
HN
N
N
O
O
Br
8
f1 (ppm)

1-(Tetrahydro-2*H*-pyran-4-yl)piperidine-4-amine (mixture of **10**·TFA and **10**·2 TFA)

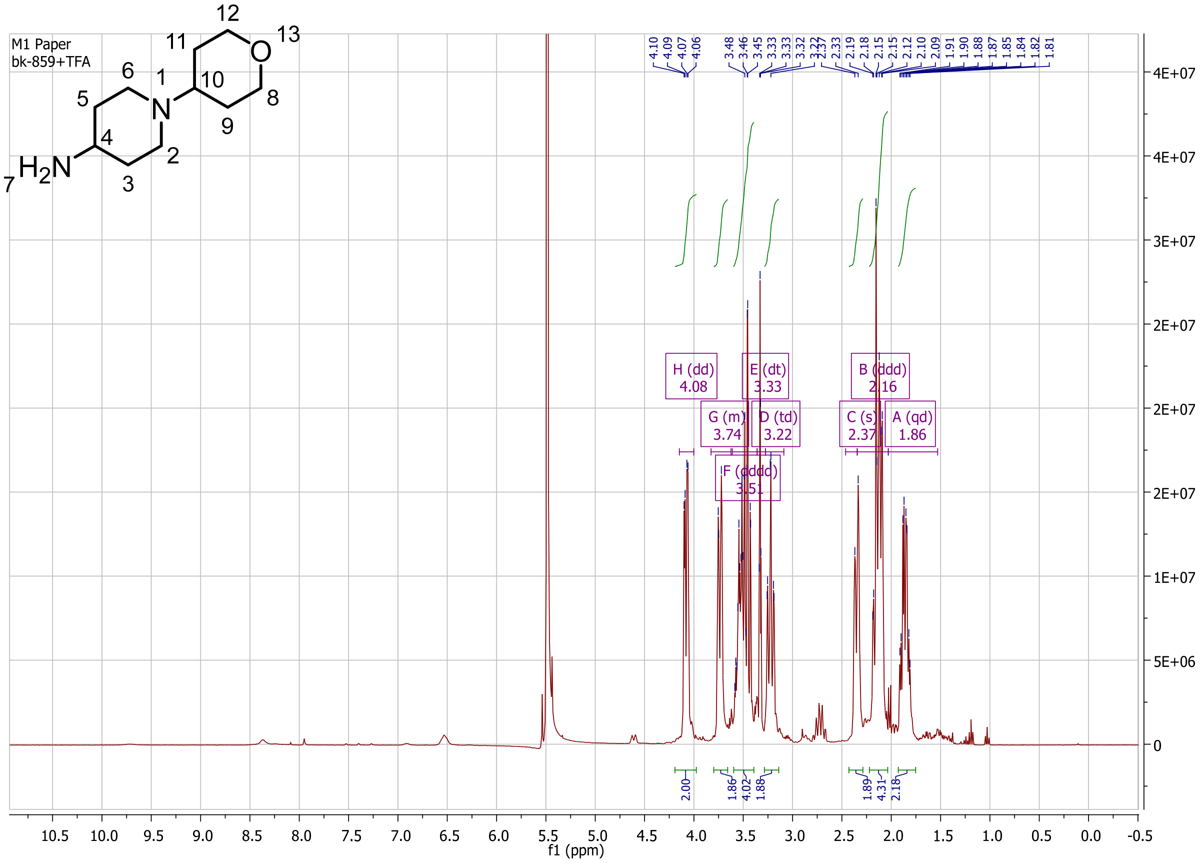

N-(4-Bromo-5-methyl-2-nitrophenyl)-1-(tetrahydro-2*H*-pyran-4-yl)piperidine-4-amine (**11b**)

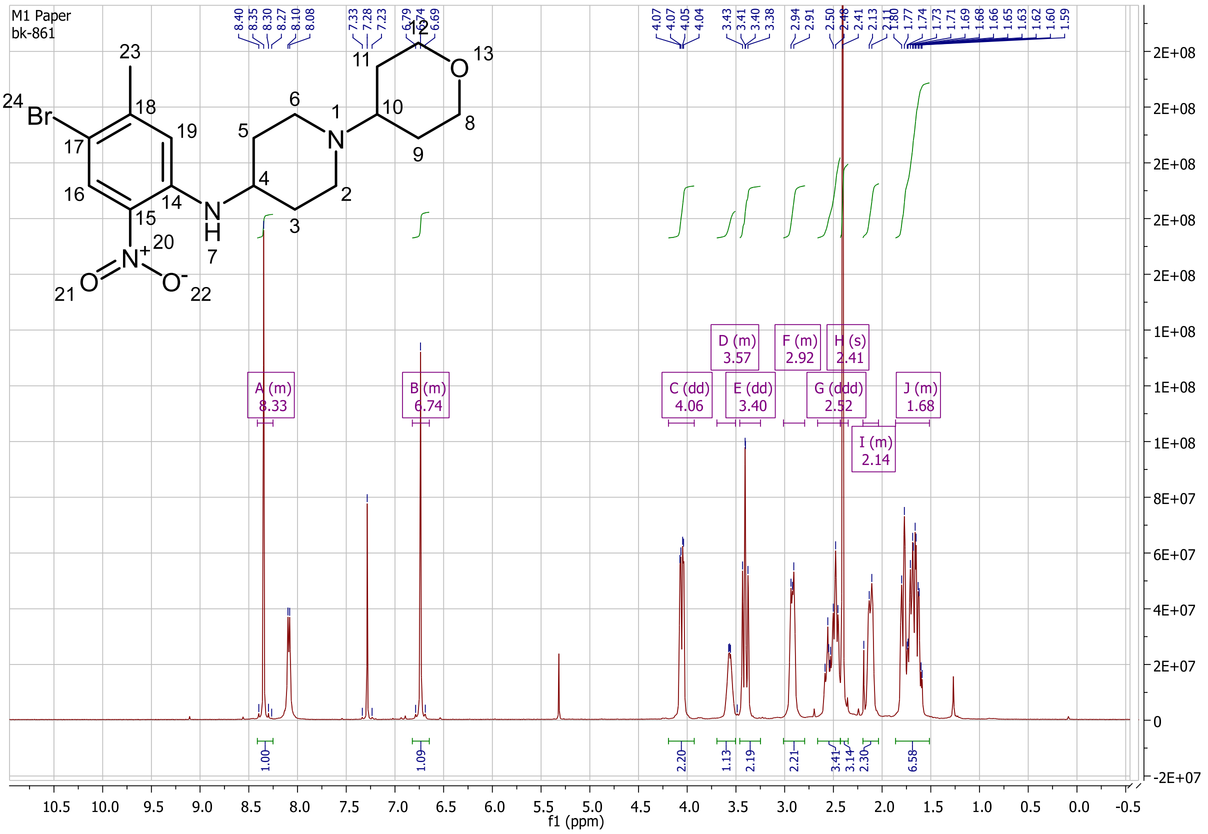

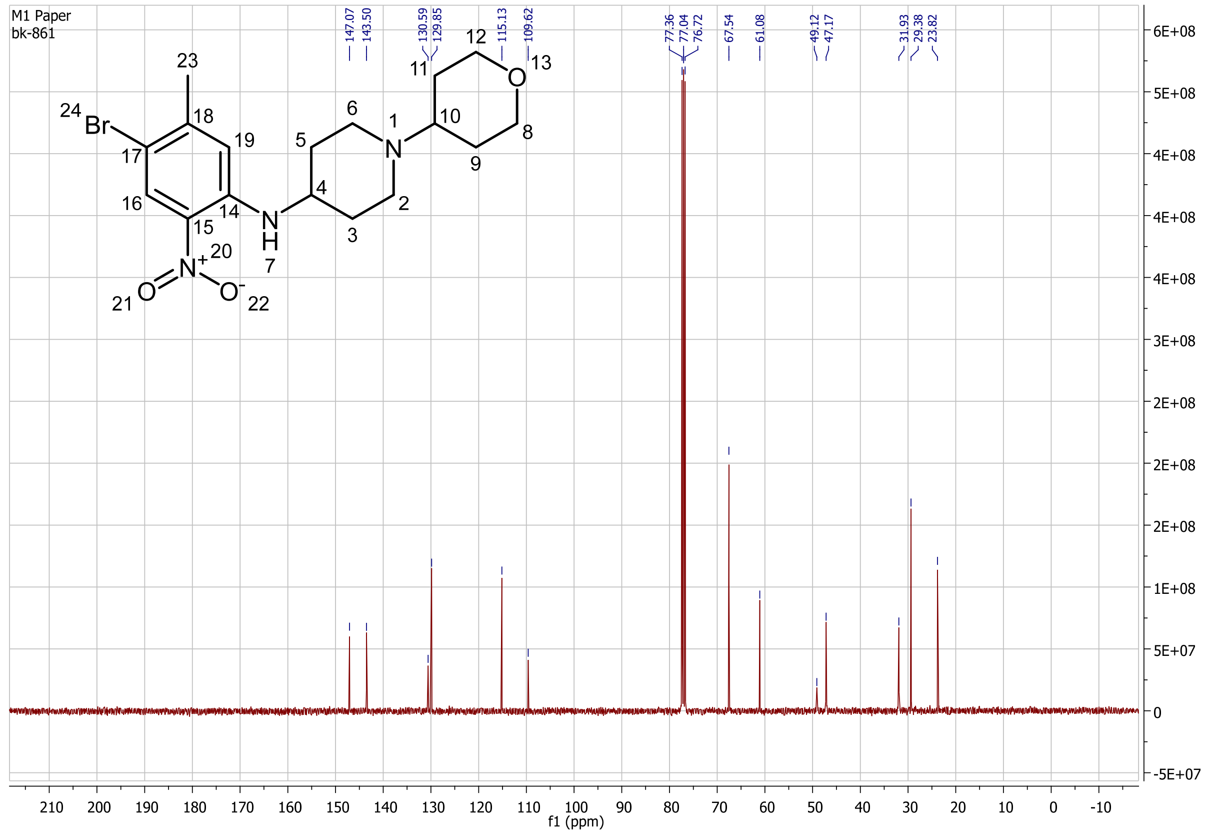

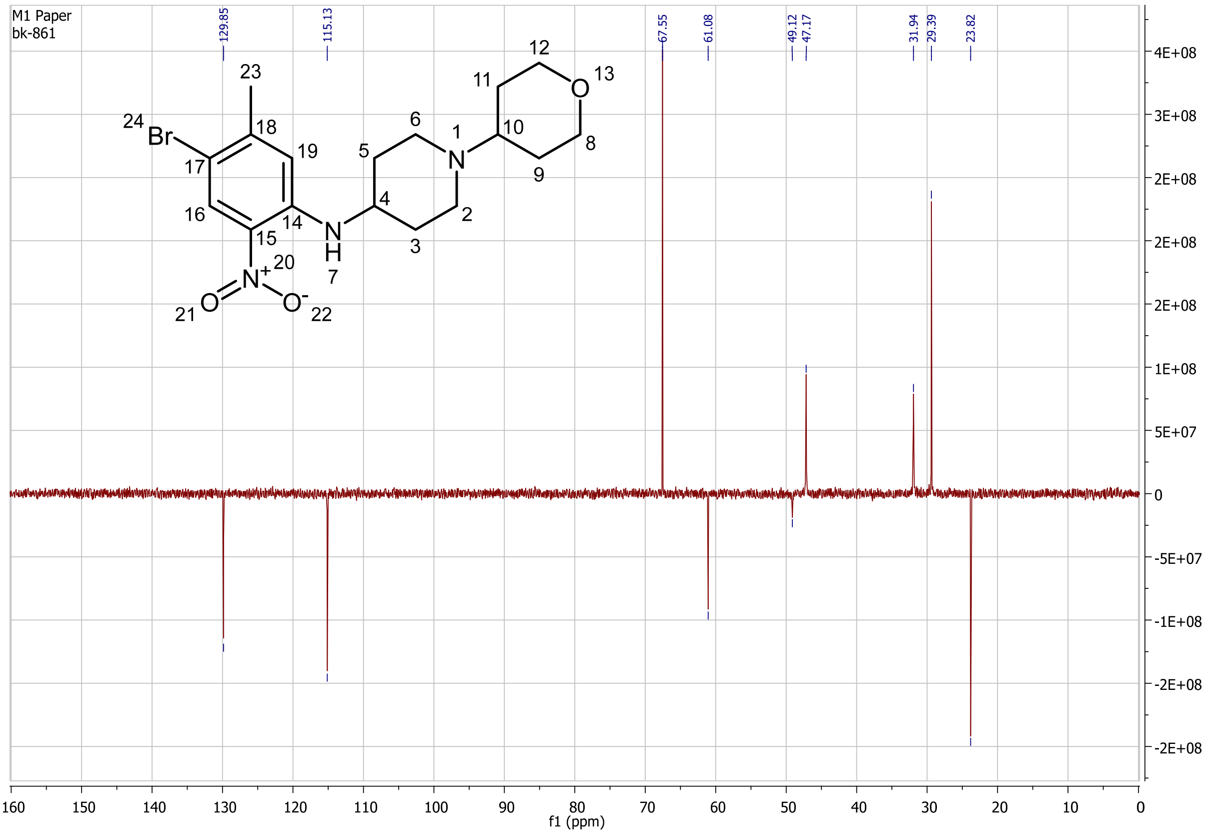

M1 Paper
bk-861
129.85
115.13
67.55
61.08
49.12
47.17
31.94
29.39
23.82
4E+08
3E+08
2E+08
2E+08
2E+08
1E+08
5E+07
0
-5E+07
-1E+08
-2E+08
-2E+08
160 150 140 130 120 110 100 90 80 70 60 50 40 30 20 10 0
f1 (ppm)

Determination of carrier amount and molar activity

The batch of [^{18}F]**1** obtained after HPLC purification (770 MBq) was left to stay for 24 h and thereafter concentrated under reduced pressure. The residue was redissolved in 20% MeCN (0.1% TFA) (1.5 mL) and an aliquot of the solution (0.5 mL) was injected into the HPLC system. The peak area was determined and the carrier amount as well as the molar activity were calculated according to the calibration curve (λ=210 nm) (Figure S1).

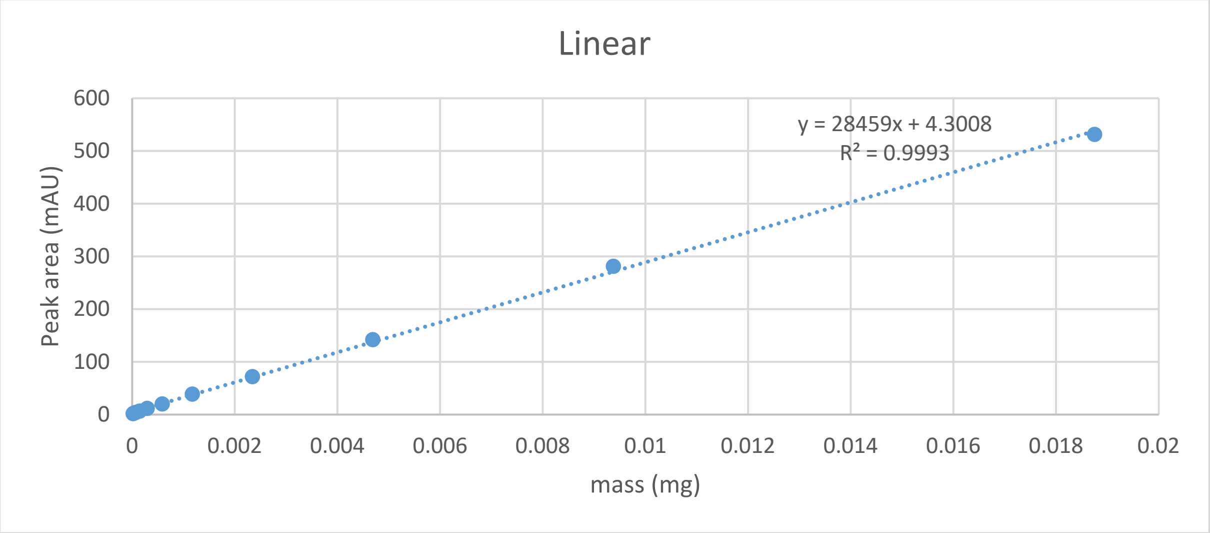

Figure S1. Calibration curve.

1/3 Batch; peak area: 83.92 mAU (corresponds to 2.8 µg). Batch; peak area: 230.21 mAU (corresponds to 8.4 µg).

Mr(**1**) = 333.4 g/mol.

Carrier amount = 25.2 nmol/batch.

Molar activity = 30.6 GBq/µmol.
